# Huayu-qutan formula ameliorates hypertrophic cardiomyopathy by regulating MAPK and HIF‑1α-signaling pathways

**DOI:** 10.1186/s13020-026-01400-5

**Published:** 2026-05-29

**Authors:** Wei Ding, Ni Zhang, Lei Xiao, Le Yu, Tian-Shi Wu, Yuan-Jie Feng, Wei Chen

**Affiliations:** Affiliated Hospital of Liaoning University of Traditional Chinese Medicine, No. 33 Beiling Street, Huanggu District, Shenyang, 110032 China

**Keywords:** Hypertrophic cardiomyopathy, Microvascular dysfunction, Fibrosis, Huayu Qutan Formula, MAPK pathway, HIF-1α

## Abstract

**Background:**

Hypertrophic cardiomyopathy (HCM) is often complicated by coronary microvascular dysfunction (CMD), which precipitates localized myocardial ischemia and metabolic disturbance, triggering compensatory cardiomyocyte hypertrophy. This hypertrophy, in turn, exacerbates microvascular insufficiency, forming a pathological feedback loop and culminating in persistent cellular stress, inflammatory infiltration, and myocardial fibrosis. This “ischemia–hypertrophy–inflammation–fibrosis” interrelationship not only drives pathological remodeling but also significantly elevates the risk of heart failure, arrhythmias, and adverse prognosis. The traditional formula Huayu Qutan Formula (HYQT), used clinically for years, has been shown to significantly enhance cardiac function in HCM patients. However, its underlying mechanisms remain poorly elucidated. This study aims to explore HYQT’s therapeutic effects on HCM and to reveal its intrinsic mechanisms.

**Methods:**

Blood-absorbed constituents of HYQT were identified using UHPLC-MS, followed by network pharmacology analysis and molecular docking to predict potential targets and key signaling pathways. An isoproterenol (ISO)-induced HCM mouse model, along with parallel in vitro injury models using H9C2 cardiomyocytes and human cardiac microvascular endothelial cells (HCMECs), was established to systematically assess the in vivo and in vitro effects of HYQT. To comprehensively evaluate HYQT’s modulation of myocardial hypertrophy, fibrosis, and angiogenesis, echocardiography, laser speckle perfusion imaging, immunohistochemistry, western blotting, reactive oxygen species (ROS) assays, and wound-healing assays were performed.

**Results:**

UHPLC-MS analysis identified 31 blood-absorbed bioactive components of HYQT. Integrated network pharmacology and molecular docking analyses identified the MAPK and HIF-1α-signaling pathways as key therapeutic targets. In ISO-induced HCM mice, HYQT significantly improved cardiac morphology and function, reduced hypertrophic markers (ANP and BNP), inhibited myocardial collagen deposition, attenuated fibrosis, restored microvascular perfusion and density, and suppressed oxidative stress (MDA and ROS) and pro-inflammatory cytokine expression (TNF-α and IL-6). Mechanistically, in vitro experiments supported a cell-type–resolved, parallel regulatory effect: in HCMECs, HYQT-containing serum enhanced migration and viability, reduced ROS accumulation, and activated HIF-1α/VEGF-mediated angiogenic signaling, thereby alleviating microvascular dysfunction; concurrently, in H9C2 cardiomyocytes, HYQT inhibited phosphorylation of ERK, p38, and JNK, restored mitochondrial membrane potential, and reduced cell death, thereby mitigating hypertrophic remodeling and its downstream fibrotic progression.

**Conclusion:**

HYQT effectively ameliorates key pathological features of HCM, including myocardial hypertrophy, fibrosis, and microvascular dysfunction. As a clinically used herbal formulation, this study provides mechanistic insight into its therapeutic effects by revealing a cell-type–resolved, myocardium–microvasculature regulatory framework. Specifically, HYQT suppresses MAPK signaling to attenuate cardiomyocyte hypertrophy while enhancing HIF-1α–mediated angiogenesis to improve the microvascular environment. These simultaneous effects are associated with mitigation of pathological cardiac remodeling, suggesting that HYQT may serve as a promising multi-target therapeutic strategy for HCM.

**Supplementary Information:**

The online version contains supplementary material available at 10.1186/s13020-026-01400-5.

## Introduction

Hypertrophic cardiomyopathy (HCM), a myocardial disorder, is characterized by asymmetric or symmetric ventricular hypertrophy, which is often accompanied by notable diastolic dysfunction [1]. Reportedly, the core pathology of HCM includes cardiomyocyte hypertrophy, disorganized arrangement, interstitial fibrosis, and cardiac microvascular dysfunction, which collectively drive severe complications such as heart failure, sudden cardiac death, and myocardial infarction [2, 3]. Present therapeutic strategies, primarily involving traditional beta-blockers and calcium channel blockers, mainly offer symptomatic relief and are ineffective for approximately 30% of patients. Furthermore, agents targeting underlying pathological mechanisms such as cardiac myosin inhibitors (e.g., mavacamten) have been shown to ameliorate outflow tract obstruction and improve cardiac function; however, high costs, potential hepatotoxicity, and restricted applicability limit their long-term utility. Metabolic modulators can optimize energy metabolism, but they cannot significantly enhance myocardial efficiency. Additionally, interventional and surgical approaches, including alcohol septal ablation, radiofrequency ablation, and septal myectomy, can alleviate obstruction or improve symptoms, but complications, high costs, and surgical risks present notable challenges. Beyond these, heart transplantation remains a viable option for end-stage cases but is constrained by donor shortage [1, 4–6]. These challenges necessitate the development of novel, safe, convenient, and efficient therapies, including potential traditional Chinese medicine (TCM) approaches, in this field in both clinical and research contexts.

In patients with HCM, coronary microvascular dysfunction (CMD) is a critical driver of myocardial ischemia. Even without macrovascular stenosis, microcirculatory deficits cause substantial perfusion defects [7, 8]. Structural changes, including luminal narrowing, vascular wall thickening, and capillary rarefaction, impair stress-induced vasodilation and blood supply in the microvascular bed [9]. These microcirculatory abnormalities trigger recurrent focal ischemia, which not only promotes myocardial fibrosis [10, 11] but is also linked to cardiac hypertrophy, creating a vicious cycle of “ischemia → hypertrophy → hypoperfusion”. Chronic ischemia further induces cellular stress and inflammation, activating fibrotic pathways and exacerbating myocardial remodeling [12]. This interconnected network of “ischemia–hypertrophy–inflammation–fibrosis” drives pathological remodeling and elevates the risk of heart failure, arrhythmias, and sudden cardiac death [11, 13, 14].

The mitogen-activated protein kinase (MAPK) pathway, including extracellular signal-regulated kinase (ERK), p38, and Janus kinase (JNK), serves as a central stress-response hub in HCM. Consequently, its aberrant activation drives pathological hypertrophy, fibroblast activation, oxidative stress, and inflammation. Notably, enhanced Rat sarcoma–MAPK–ERK signaling has been shown to promote cardiomyocyte hypertrophy, whereas pharmacological MAPK kinase inhibition reverses HCM-like phenotypes [15–17]. These observations position MAPK signaling as a promising therapeutic target in HCM. HCM progression is further exacerbated by energy supply–demand imbalances and relative hypoxia due to myocardial thickening and microvascular dysfunction. Hypoxia-inducible factor-1α (HIF-1α) acts as a master regulator of adaptive hypoxia responses, governing angiogenesis and oxygen homeostasis. However, in HCM's chronic pathological environment, HIF-1α signaling often becomes dysregulated or depleted, impairing microvascular development and worsening myocardial injury [18, 19]. Together, excessive MAPK activation and HIF-1α dysregulation may form a vicious cycle of ischemia, hypertrophy, inflammation, and fibrosis. Hence, optimal therapies need to move beyond the simple “up-” or “down-regulation” paradigm and aim to simultaneously suppress pathological MAPK signaling and restore HIF-1α's protective roles.

**Scheme 1:**
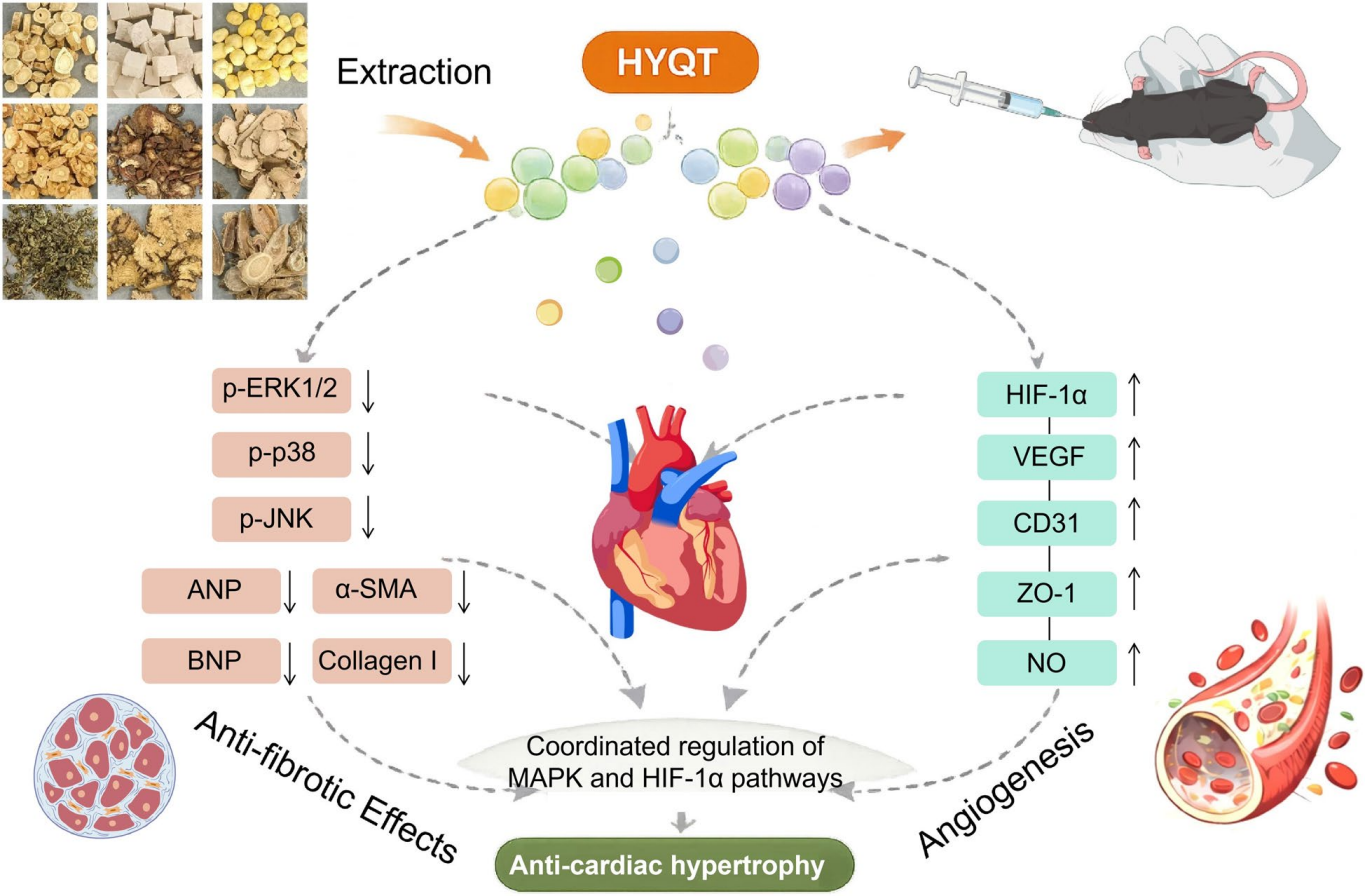
Schematic of a possible molecular mechanism by which HYQT improves HCM

Huayu Qutan Formula (HYQT), composing *Astragalus membranaceus*, *Codonopsis pilosula*, *Gynostemma pentaphyllum*, *Poria cocos*, *Salvia miltiorrhiza*, *Ligusticum chuanxiong*, *Pinellia ternata*, *Acorus tatarinowii*, and *Curcuma aromatica* (Table 1), is a patented, hospital-prepared TCM formulation from the Cardiovascular Department of Liaoning University of Traditional Chinese Medicine Affiliated Hospital. In a clinical setting, it reduces myocardial fibrosis and collagen deposition, improves microcirculatory perfusion in affected myocardial regions, promotes cardiac function recovery, and lowers major adverse cardiovascular events. However, its precise mechanisms in HCM remain elusive. This study aimed to elucidate HYQT’s cardioprotective mechanisms and role in alleviating myocardial microvascular dysfunction by investigating its regulatory effects on the MAPK/HIF-1α-signaling pathway (Scheme1). Herein, both in vitro and in vivo models were employed to explore the molecular mechanisms underlying HYQT-mediated amelioration of HCM. This study provides an experimental basis for further exploration of TCM-based prevention and treatment strategies for HCM.

**Table 1:** Composition of HYQT

Pharmaceutical name	Botanical name	Chinese name	Amount (g)	Physical diagram
Astragali Radix	Astragalus membranaceus (Fisch.) Bge. var. mongholicus (Bge.) Hsiao	黄芪	20	
Codonopsis Radix	Codonopsis pilosula (Franch.) Nannf	党参	15	
Gynostemmatis Herba	Gynostemma pentaphyllum (Thunb.) Makino	绞股蓝	15	
Poria	Poria cocos (Schw.) Wolf	茯苓	15	
Salviae Miltiorrhizae Radix et Rhizoma	Salvia miltiorrhiza Bunge	丹参	15	
Chuanxiong Rhizoma	Ligusticum chuanxiong Hort	川芎	10	
Pinelliae Rhizoma Praeparatum	Pinellia ternata (Thunb.) Breit	法半夏	10	
Acori Tatarinowii Rhizoma	Acorus tatarinowii Schott	石菖蒲	10	
Radix Curcumae	Curcuma wenyujin Y.H.Chen et C.Ling、Curcuma longa L.、Curcuma kwangsiensis S.G.Lee et C.F.Liang or Curcuma phaeocaulis Val	郁金	10	

The herb names are from The Pharmacopoeia of the People’s Republic of China (2020 Edition)

## Materials and methods

### Reagents

Astragalus membranaceus (Fisch.) Bge. (Batch No: A240918), Codonopsis pilosula (Franch.) Nannf. (Batch No: A240821), Gynostemma pentaphyllum (Thunb.) Makino (Batch No: 240501), Poria cocos (Schw.) Wolf (Batch No: A230904), Salvia miltiorrhiza Bge. (Batch No: A240826), Ligusticum chuanxiong Hort. (Batch No: A240931), Pinellia ternata (Thunb.) Breit. (Batch No: A241027), Acorus tatarinowii Schott (Batch No: GT240106073), Curcuma aromatica Salisb. (Batch No: 240601), All medicinal materials were purchased from the School of Pharmacy, Liaoning University of Traditional Chinese Medicine (Dalian, China), and authenticated by the Department of Chinese Materia Medica Identification, Liaoning University of Traditional Chinese Medicine, according to the standards of the Chinese Pharmacopoeia (2020 edition).

Assay kits for atrial natriuretic peptide (ANP), B-type natriuretic peptide (BNP), and N-terminal pro-B-type natriuretic peptide (NT-proBNP) were purchased from GeneMay Biotechnology Co., Ltd. (Wuhan, China). The following reagents were obtained from Solarbio Science & Technology Co., Ltd. (Beijing, China): Biochemical assay kits for Total Superoxide Dismutase (T-SOD), Malondialdehyde (MDA), Catalase (CAT), Nitric Oxide (NO), Total Nitric Oxide Synthase (T-NOS), Inducible Nitric Oxide Synthase (iNOS), Oxidized Glutathione (GSSG), and Total Glutathione (T-GSH); Enzyme-Linked Immunosorbent Assay (ELISA) kits for Tumor Necrosis Factor-alpha (TNF-α), Interleukin-1beta (IL-1β), Interleukin-6 (IL-6), and Interleukin-10 (IL-10); Liver and kidney function assay kits (for detecting albumin (ALB), alkaline phosphatase (ALP), alanine aminotransferase (ALT), aspartate aminotransferase (AST), urea (UREA), and creatinine (CREA)) were obtained from Beijing Applygen Technologies Inc. (Beijing, China). Cell Counting Kit-8 (CCK-8) was purchased from Shenyang Obiory Biotechnology Co., Ltd. (Shenyang, China). Primary antibodies against α-smooth muscle actin (α-SMA), Collagen I, Caspase-3, CD31, endothelial nitric oxide synthase (eNOS), vascular endothelial growth factor (VEGF), and zonula occludens-1 (ZO-1), as well as secondary antibodies (FITC-conjugated IgG, Cy3-conjugated IgG, and HRP-biotin-conjugated IgG), were all acquired from Boster Biological Technology Co., Ltd. (Wuhan, China). Antibodies targeting extracellular signal-regulated kinase 1/2 (ERK1/2), p38 mitogen-activated protein kinase (p38 MAPK), c-Jun N-terminal kinase (JNK), phosphorylated ERK1/2 (p-ERK1/2), phosphorylated p38 MAPK (p-p38 MAPK), phosphorylated JNK (p-JNK), and hypoxia-inducible factor 1-alpha (HIF-1α) were purchased from Affinity Biosciences (Jiangsu, China). Isoproterenol, PD98059, SP600125, SB203580, YC-1 and bisBenzimide H 33342 trihydrochloride (Hoechst 33,342) were obtained from Aladdin Biochemical Technology Co., Ltd. (Shanghai, China). All other chemicals and reagents used in this study were of analytical grade.

### Animal study

The rat cardiomyocyte cell line H9C2 was purchased from iCell Bioscience Inc. (Shanghai, China). Human Cardiac Microvascular Endothelial Cells (HCMECs) were purchased from Shanghai Zhong Qiao Xin Zhou Biotechnology Co., Ltd. (Shanghai, China). Male Sprague–Dawley rats (180–220 g) used for serum preparation were obtained from Liaoning Changsheng Biotechnology Co., Ltd. (Benxi, China) and were orally administered HYQT twice daily for seven consecutive days. Eight-week-old male C57BL/6 J mice (specific pathogen-free [SPF] grade) were purchased from Liaoning Changsheng Biotechnology Co., Ltd. (Benxi, China). The mice were housed in a temperature-controlled facility (20–25 °C) under a 12-h light/dark cycle with free access to food and water. All surgical procedures and euthanasia were performed under anesthesia induced by intraperitoneal injection of sodium pentobarbital (40 mg/kg). Blood collection was performed under deep anesthesia prior to euthanasia. Euthanasia was confirmed by cervical dislocation after ensuring the absence of pedal reflexes. All experimental procedures were performed in accordance with the guidelines of the Experimental Animal Ethics Committee of Liaoning University of Traditional Chinese Medicine and were approved by this committee (Approval No.: 210000620250229).

### Extraction of the HYQT decoction

The herbal materials were weighed according to the proportions listed in Table 1 and soaked in ten volumes of distilled water for 30 min. The mixture was then brought to a boil and decocted three times for 2 h, 2 h, and 1.5 h, respectively. The combined extracts were filtered and concentrated under reduced pressure to yield a final concentration equivalent to 1.2 g of raw herbs per milliliter. The concentrate was lyophilized and stored in a desiccator until use. The low, medium, and high doses of HYQT used in the mouse study (8, 16, and 32 g/kg, respectively) were determined based on the body surface area normalization method for dose translation from the clinical human equivalent dose, and were selected to explore a potential dose–response relationship.

### Preparation of drug‑containing serum

Male Sprague–Dawley (SD) rats (weighing 180–220 g), purchased from Liaoning Changsheng Biotechnology Co., Ltd., were orally administered HYQT at a dose of 10 g/kg twice daily for seven consecutive days. One to two hours after the final dose, blood was aseptically collected from the abdominal aorta under anesthesia. Samples were allowed to clot at room temperature for 30–60 min, then centrifuged at 3,000 rpm for 15 min at 4 °C. The serum supernatant was heat‑inactivated in a 56 °C water bath for 30 min, sterile‑filtered through a 0.22 µm membrane, aliquoted, and stored at − 80 °C. Blank serum was prepared simultaneously using the same protocol in control rats. After blood collection, the rats were immediately euthanized.

### Establishment of the animal model

Thirty-six male C57BL/6 J mice (8 weeks old, SPF grade) were randomly assigned to six groups (n = 6 each): (1) Control (no treatment); (2) Model group (isoproterenol [ISO] 5 mg/kg subcutaneously, once daily for 2 weeks); (3) Low-dose HYQT group (ISO + HYQT 8 g/kg); (4) Medium-dose HYQT group (ISO + HYQT 16 g/kg); (5) High-dose HYQT group (ISO + HYQT 32 g/kg); and (6) Positive control group (ISO + nicorandil 5 mg/kg). Following the 2-week ISO induction period to establish the HCM model, the HYQT decoction and nicorandil treatments were administered for 4 weeks according to the administration time and concentration set based on the preliminary experiment results. Body weight was recorded weekly. At the end of treatment, cardiac function was assessed by electrocardiography, echocardiography, and laser speckle imaging. Mice were then anesthetized; blood was collected, and hearts were excised, photographed, and weighed for the calculation of heart weight to body weight ratio. Portions of cardiac tissue were fixed in 4% paraformaldehyde for histology, and the remainder was snap‑frozen at − 80 °C for subsequent Western blot analysis.

### Biochemical analyses

Serum levels and activities of atrial natriuretic peptide (ANP), B‑type natriuretic peptide (BNP), N‑terminal pro‑BNP (NT‑proBNP), total superoxide dismutase (T‑SOD), malondialdehyde (MDA), catalase (CAT), nitric oxide (NO), total nitric oxide synthase (T‑NOS), inducible nitric oxide synthase (iNOS), oxidized glutathione (GSSG), total glutathione (T-GSH), tumor necrosis factor‑α (TNF‑α), interleukin IL‑1β, IL‑6, IL‑10, albumin (ALB), alkaline phosphatase (ALP), alanine aminotransferase (ALT), aspartate aminotransferase (AST), urea, and creatinine (CREA) were measured using commercial assay kits, strictly following the manufacturers’ instructions.

### Cell culture and treatment

H9C2 cells were cultured in DMEM supplemented with 10% fetal bovine serum (FBS) and 1% penicillin–streptomycin (P/S), while human cardiac microvascular endothelial cells (HCMECs) were maintained in HCMEC-specific medium. All cells were incubated at 37 °C in a humidified atmosphere containing 95% air and 5% CO_2_ and subcultured every 2–3 days. For drug intervention, cells were divided into the following groups: control, model (ISO), low-, medium-, and high-dose HYQT-containing serum treatment groups (ISO + HYQT at final serum concentrations of 10%, 20%, and 40%, respectively), and a positive control group (ISO + nicorandil). Preliminary experiments confirmed that blank serum (from saline-gavaged rats) at corresponding concentrations did not significantly alter the ISO-induced injury response. The HYQT concentrations were selected based on preliminary cytotoxicity assays, which showed that cells maintained normal morphology and viability at 40%. Therefore, 10%, 20%, and 40% of the drug-containing serum were chosen to represent low, medium, and high doses, respectively. The positive control, nicorandil, was used at 50 μM, a concentration chosen according to previous studies and validated by our pilot experiments. After treatment, all groups were incubated for 24 h, followed by subsequent analyses.

### Wound healing assay

HCMECs were seeded in 6-well plates at a density of 5 × 10^5^ cells/mL and cultured for 12 h until approximately 90% confluency. Three parallel scratches were generated in each well using a sterile 200 μL pipette tip. After washing three times with pre-cooled PBS to remove detached cells, the medium was replaced with DMEM containing 1% fetal bovine serum (FBS). The cells were divided into the following treatment groups: Blank control group; Model group (treated with ISO at a final concentration of 1 mM); HYQT treatment groups (following 48-h exposure to ISO (1 mM), the medium was replaced with fresh medium containing 10%, 20%, or 40% HYQT-containing serum for an additional 48 h); Positive control group (after 48-h ISO (1 mM) treatment, the medium was replaced with fresh medium containing 50 μM nicorandil for another 48 h). Cell migration images were captured at 0, 12, and 24 h after scratch formation using phase-contrast microscopy at predetermined fields. The wound areas were quantified using ImageJ software, and the cell migration rate was calculated as follows:

$${\text{Cell migration rate }}\left( \% \right)\, = \,\left[ {\left( {{\text{Wound area at }}0 {\mathrm{h}}\, - \,{\text{Wound area at specific time point}}} \right)/{\text{Wound area at }}0 {\mathrm{h}}} \right]\, \times \,{1}00.$$

To distinguish the effect on cell migration from potential influences on cell viability, the cytotoxicity of the treatments was assessed in parallel using the Live/Dead Cell Staining Kit under identical culture and treatment conditions.

### Intracellular reactive oxygen species (ROS) detection

HCMECs and H9C2 cells were seeded in 96-well plates at a density of 1 × 10^4^ cells/mL, respectively, and allowed to adhere. To establish the cellular model, all groups except the control were treated with ISO at a final concentration of 1 mM for 48 h. After the modeling period, the culture medium was replaced, and the cells were divided into the following groups for a subsequent 48-h intervention: (1) Blank control group: normal culture medium; (2) Model group: fresh medium containing ISO (1 mM); (3) HYQT-containing serum groups: fresh medium containing 10%, 20%, or 40% HYQT-containing serum; (4) Positive control group: fresh medium containing 50 μM Nicorandil. Following the treatment period, cells were washed with PBS and incubated with DCFH-DA fluorescent probe in the dark for 20 min, followed by nuclear staining with DAPI for 15 min. After final PBS rinses, fluorescent images were captured, and intracellular ROS levels were quantified by measuring DCF fluorescence intensity using Image J software.

### Live/dead cell staining

The treatment procedure followed that of the ROS detection assay. After 48 h of exposure to drug-containing serum, cells were washed with PBS and incubated with live/dead staining solution in the dark for 30 min. After washing, fluorescence microscopy was performed. The number of live (green) and dead (red) cells was quantified using Image J software.

$${\text{Cell death rate }}\left( \% \right)\, = \,\left[ {{\text{Number of dead cells}}/\left( {{\text{Number of live cells}}\, + \,{\text{Number of dead cells}}} \right)} \right]\, \times \,{1}00.$$

### Measurement of mitochondrial membrane potential level

The treatment procedure was consistent with that of the ROS detection experiment. After 24 h of treatment with medicated serum, the cells were washed with PBS and co-incubated with a fluorescent probe for mitochondrial membrane potential detection in the dark for 15 min. Following washing, images were immediately captured using an inverted fluorescence microscope. Red fluorescence (J-aggregates) indicates normal mitochondrial membrane potential, while green fluorescence (J-monomers) indicates a decrease in membrane potential. The red-to-green fluorescence ratio (Red/Green) was calculated using Image J to quantitatively assess the ΔΨm level.

### Cell mechanism validation experiments

In the mechanism validation experiments, H9C2 cardiomyocytes and HCMEC endothelial cells were treated with MAPK and HIF-1α pathway inhibitors, respectively. For the MAPK pathway inhibition experiment, H9C2 cells were divided into the following groups: blank control, model (ISO), high-dose HYQT (H-HYQT), and HYQT combined with the corresponding MAPK inhibitors (PD98059, SP600125, and SB203580), with each inhibitor used at a concentration of 10 μM. After 24 h of treatment, cells were analyzed by immunofluorescence to detect the total and phosphorylated protein levels of ERK1/2, JNK, and p38 MAPK, and the semi-quantitative analysis was performed to calculate the ratio of phosphorylated MAPK to total MAPK. Flow cytometry was used to measure intracellular ROS levels to assess the effect of MAPK inhibition on ROS, and ANP and BNP levels were also measured. For the HIF-1α pathway inhibition experiment, HCMEC cells were similarly divided into the following groups: blank control, model (ISO), high-dose HYQT (H-HYQT), and HYQT combined with the HIF-1α inhibitor YC-1, with YC-1 used at a concentration of 10 μM. After 24 h of treatment, Western blot analysis was performed to detect the protein expression of HIF-1α, VEGF, and CD31, and flow cytometry was used to measure ROS levels to assess the effect of HIF-1α inhibition on ROS. Additionally, cell viability was evaluated using the CCK-8 assay to assess the impact of HYQT and HIF-1α inhibition on endothelial cell survival.

### Hematoxylin and eosin (H&E) staining

Paraffin-embedded heart tissue sections were routinely deparaffinized and rehydrated. Sections were stained with hematoxylin for 20 min, differentiated for 2 s, blued for 5 min, and counterstained with eosin for 2 min. Finally, slides were mounted using neutral resin and examined under a light microscope for histopathological evaluation.

### Masson’s trichrome staining

Paraffin heart sections were incubated overnight at room temperature in 2.5% potassium dichromate solution, followed by 30 min incubation at 65 °C. After rinsing, sections were stained with Weigert’s iron hematoxylin for 1 min, differentiated in 1% hydrochloric acid–ethanol for 1 min, and washed. Sections were then stained with ponceau-acid fuchsin for 6 min, treated with 1% PBS for 1 min, and counterstained with 2.5% aniline blue for 20 s. After a final 10 s soak in 1% acetic acid, sections were dehydrated through graded ethanol, cleared with xylene, and mounted with neutral resin.

### Sirius red staining

Following deparaffinization and PBS washing, heart sections were stained with Sirius Red solution for 10 min. Sections were dehydrated with absolute ethanol, cleared with xylene for 5 min, and mounted with neutral resin. Collagen fibers were visualized and analyzed under a light microscope.

### Immunohistochemical (IHC)/immunofluorescence (IF) staining

Paraffin-embedded heart tissue sections were deparaffinized in xylene and rehydrated through a graded ethanol series. Endogenous peroxidase activity was blocked using 3% hydrogen peroxide (H_2_O_2_) for 15 min, followed by antigen retrieval in 0.1 M sodium citrate buffer. Sections were then blocked with 5% BSA at room temperature for 1 h and incubated with primary antibodies against α-SMA, Caspase-3, Collagen I, eNOS, VEGF, CD31, and ZO-1 (1:200 dilution) for 1 h at room temperature. After washing, sections were incubated for 1 h with Cy3-, FITC-, or HRP-conjugated secondary antibodies (1:200 dilution). Nuclei were counterstained with DAPI or DAB for 10 min. The stained sections were observed and imaged under a fluorescence or bright-field microscope as appropriate.

### UHPLC-Q-TOF MS analysis

One gram of powdered herbal material was accurately weighed and placed into a 50 mL stoppered conical flask. Twenty milliliters of 50% methanol–water solution was added, and the flask was sealed and weighed. Ultrasonic extraction was performed for 30 min at 500 W and 100 kHz. After cooling to room temperature, the weight loss was replenished with 50% methanol–water to the original weight. The mixture was thoroughly vortexed and filtered through a 0.22 µm microporous membrane. The resulting filtrate was collected as the test solution.

### Serum sample preparation

Whole blood samples were centrifuged at 3500*g* for 15 min at 4 °C. The supernatant (serum) was carefully collected. A defined volume of serum was mixed with five volumes of pre-chilled methanol\:acetonitrile (1:5, v/v), vortexed for 30 s, and incubated at − 20 °C for 30 min to precipitate proteins. After centrifugation at 12,000*g* for 15 min at 4 °C, the supernatant was collected and evaporated under a gentle stream of nitrogen. The residue was reconstituted in 50% methanol–water, vortexed, filtered through a 0.22 µm membrane, and the filtrate was subjected to UHPLC-Q-TOF MS analysis.

### Chromatographic and mass spectrometric conditions

Chromatographic separation was achieved using a Waters ACQUITY UPLC HSS T3 column (100 mm × 2.1 mm, 1.6 µm). The mobile phases consisted of 0.1% formic acid in water (A) and acetonitrile (B). The gradient elution was as follows: 0–56 min, 5% → 95% B; 56–58 min, 95% B; 58–58.01 min, 95% → 5% B; 58.01–60 min, 5% B. The column temperature was maintained at 40 °C, the flow rate was 0.3 mL/min, and the injection volume was 5 µL.

Mass spectrometry was performed on a SCIEX X500R Q-TOF instrument with an electrospray ionization (ESI) source operating in both positive and negative ion modes with alternate scanning. Data acquisition was conducted using information-dependent acquisition (IDA) mode. Full-scan MS spectra were recorded in the m/z range of 100–1500, with MS/MS spectra triggered based on pre-defined criteria. Source parameters were as follows: ion source temperature 500 °C; ion spray voltage + 5500 V (positive mode)/− 4500 V (negative mode); curtain gas 30 psi; ion source gas 1 and gas 2 both at 50 psi. Collision energy (CE) was set to 40 V, and collision energy spread (CES) was ± 10 V.

### Network pharmacology analysis

#### Identification of HYQT core targets

Blood-absorbed constituents identified by UHPLC-Q-TOF MS were uploaded to the PubChem database ([https://pubchem.ncbi.nlm.nih.gov/](https://pubchem.ncbi.nlm.nih.gov/)) to retrieve their SMILES identifiers. These were then input into the SwissTargetPrediction database ([http://www.swisstargetprediction.ch/](http://www.swisstargetprediction.ch/)) to predict potential targets with a probability > 0. The predicted targets were subsequently standardized and deduplicated using the UniProt database ([https://www.uniprot.org/](https://www.uniprot.org/)).

#### Identification of common targets between HYQT and HCM

Using “Hypertrophic cardiomyopathy” as the keyword, disease-related targets were retrieved from GeneCards ([https://genealacart.genecards.org/](https://genealacart.genecards.org/)), OMIM ([https://www.omim.org/](https://www.omim.org/)), and TTD ([http://db.idrblab.net/ttd/](http://db.idrblab.net/ttd/)). After deduplication, the disease-related targets were intersected with HYQT-related targets to identify shared genes potentially involved in HCM treatment. Venn diagrams were generated using Draw Venn Diagram to visualize overlapping targets.

#### Construction of the drug–compound–target–disease network

Identified active compounds and their intersecting gene targets were imported into Cytoscape 3.9.1 to construct a comprehensive drug–compound–target–disease network. This model visually illustrates the potential mechanisms by which HYQT components act on HCM-related targets.

#### Protein–protein interaction (PPI) network and core target screening

Shared targets between HYQT and HCM were uploaded to the STRING database to construct a PPI network. Isolated nodes were removed, and network topology was analyzed in Cytoscape 3.9.1. Key topological parameters, including betweenness centrality, closeness centrality, and degree, were calculated using the Network Analyzer plugin. Targets with values above the median were defined as potential core targets and ranked by degree for prioritization.

#### GO and KEGG enrichment analyses

Potential core targets were subjected to Gene Ontology (GO) functional annotation and Kyoto Encyclopedia of Genes and Genomes (KEGG) pathway enrichment using the DAVID 6.8 database, restricted to *Homo sapiens*. Statistically significant terms (*P* ≤ 0.05) were selected, and the top 10 GO terms and top 25 KEGG pathways were visualized using bar plots.

#### Molecular docking

The 3D structures of HYQT core active compounds were downloaded from the PubChem database and subjected to energy minimization using ChemBio3D Ultra 14.0 (RMS gradient threshold: 0.001), then saved in MOL2 format. Receptor proteins—MAPK1 (PDB ID: 2Y9Q), HIF-1α (PDB ID: 4H6J), and P38 MAPK (PDB ID: 1A9U)—were obtained from the RCSB PDB database. Water molecules and co-crystallized ligands were removed using PyMOL 2.3.0.

Hydrogen atoms were added, Gasteiger charges were calculated, and both ligands and receptors were converted to PDBQT format using AutoDockTools 1.5.6. Binding pockets were predicted using POCASA 1.1. Molecular docking was performed using AutoDock Vina 1.1.2 with a uniform search space of 60 × 60 × 60 Å^3^ and grid spacing of 0.375 Å. Exhaustiveness was set to 10. The grid centers for ERK1/2, HIF-1α, and P38 MAPK were set at (50.5, 26.8, 7.6), (18.1, − 9.3, − 28.7), and (3.6, 16.8, 30.4), respectively. The docking results were visualized and analyzed using PyMOL 2.3.0 to explore binding modes and interaction profiles.

#### Western blot analysis

Total proteins were extracted from mouse heart tissues, HCMECs, and H9C2 cells and quantified using a BCA protein assay. Equal amounts of protein (20 μg per sample) were separated by SDS–polyacrylamide gel electrophoresis (SDS–PAGE) and transferred onto polyvinylidene difluoride (PVDF) membranes (Bio-Rad, USA). The membranes were blocked with 5% non-fat dry milk and incubated overnight at 4 °C with primary antibodies against ERK1/2, p38 MAPK, JNK, p-ERK1/2, p-p38 MAPK, p-JNK, CD31, VEGF, HIF-1α, and GAPDH (all at 1:2000 dilution). After washing three times with TBST, the membranes were incubated with horseradish peroxidase (HRP)-conjugated secondary antibodies (1:1000) at room temperature for 1 h. Protein bands were visualized using enhanced chemiluminescence (ECL) reagents, and band intensities were semi-quantified using Image J software. Target protein expression levels were normalized to β-actin and subjected to statistical analysis.

#### Statistical analysis

All statistical analyses were performed using GraphPad Prism 9.0 software. Data are presented as mean ± standard deviation (SD). One-way analysis of variance (ANOVA) followed by Bonferroni’s or Dunnett’s post hoc test was used for multiple group comparisons. For comparisons between two groups, Student’s *t*-test was applied. A *P*-value of less than 0.05 was considered statistically significant.

## Results

### HYQT mitigates ISO‑induced injury in HCMEC cells

**Fig. 1:**
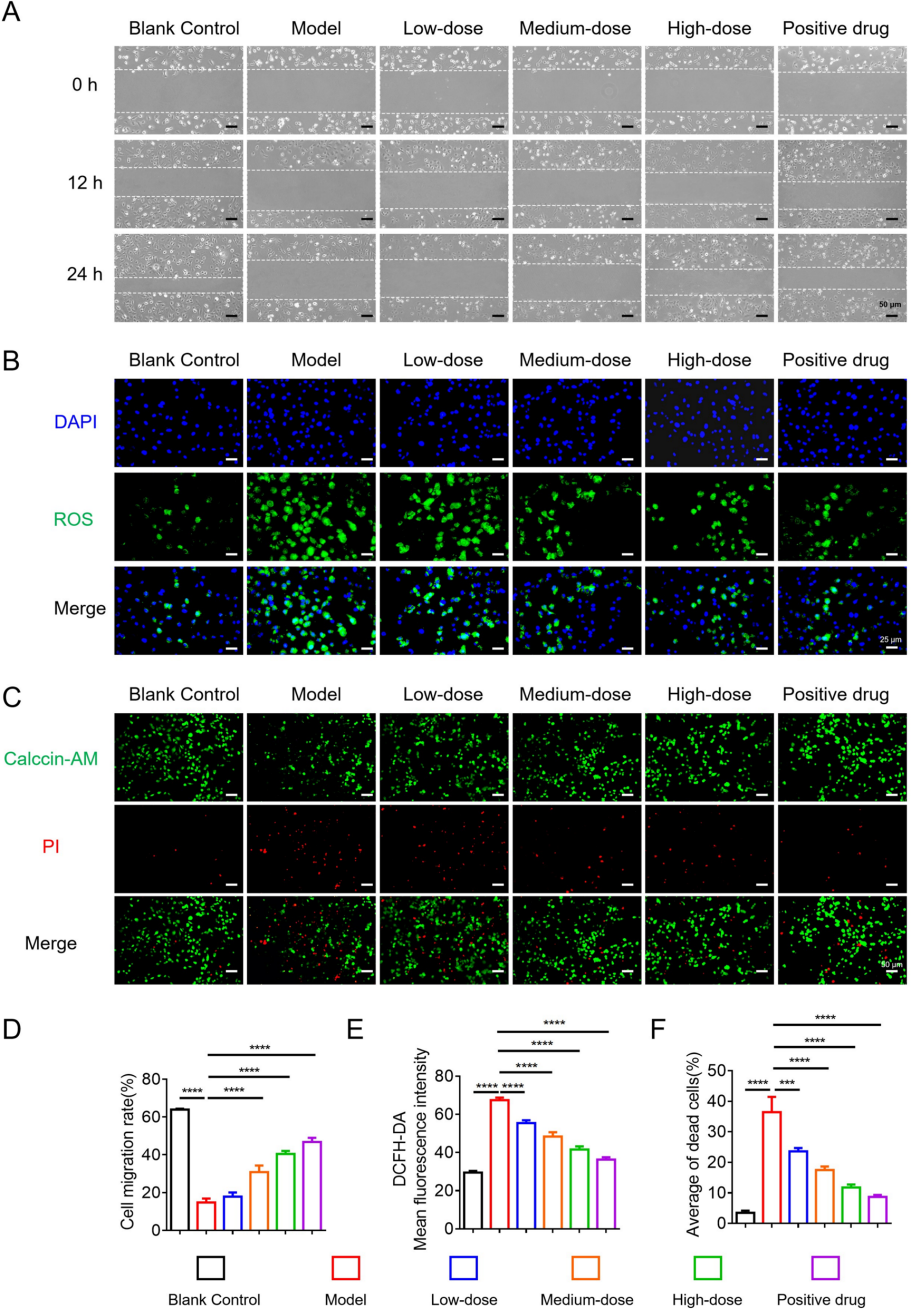
Effects of HYQT on HCMECs migration, ROS production, and viability in an in vitro model. **A** Representative images of scratch wound healing assay at 0 h, 12 h, and 24 h in different groups (scale bar = 50 μm). **B** Staining for ROS detection (DAPI for nuclear staining, green for ROS; scale bar = 25 μm). **C** Live/dead cell staining (Calcein-AM for live cells, green; PI for dead cells, red; scale bar = 50 μm). **D** Quantitative analysis of cell migration rate. **E** Mean fluorescence intensity of DCFH-DA (a probe for ROS). **F** Average percentage of dead cells. Data are presented as mean ± SD (n = 3). ^*^P < 0.05, ^**^P < 0.01, ^***^P < 0.001, ^****^P < 0.0001

In a scratch-wound assay, the migratory capacity of HCMECs was significantly impaired in the ISO-treated group compared to the control. Treatment with HYQT at low, medium, and high concentrations, as well as with nicorandil, markedly enhanced cell migration at both 12 and 24 h post-injury. These findings indicate that HYQT effectively promotes endothelial repair and may contribute to the mitigation of microvascular damage (Fig. 1A, D).

DCFH-DA fluorescence staining revealed a substantial increase in reactive oxygen species (ROS) production in ISO-treated HCMECs, as indicated by intensified green fluorescence. HYQT treatment led to a dose-dependent reduction in ROS levels at all tested concentrations, demonstrating its antioxidant effect (Fig. 1B, E).

Live/dead cell staining showed a decreased number of viable (green) cells and an increased number of non-viable (red) cells in the ISO group, indicating cytotoxicity. HYQT administration significantly improved cell viability in a dose-dependent manner, evidenced by increased green fluorescence and reduced red fluorescence. The high-dose HYQT group exhibited the most favorable viability profile. These results demonstrate that HYQT protects endothelial cells from ISO-induced damage and promotes cell survival (Fig. 1C, F).

Overall, HYQT significantly enhances HCMEC migration, attenuates oxidative stress, and improves endothelial cell viability in an ISO-induced injury model. These effects are dose-dependent and closely resemble the protective actions of established endothelial therapeutics such as nicorandil. Collectively, the data support the therapeutic potential of HYQT in promoting endothelial function and preserving microvascular integrity in the context of HCM.

### Evaluation of HYQT in ameliorating ISO-induced injury in H9C2 cells

#### Detection of ROS levels

**Fig. 2:**
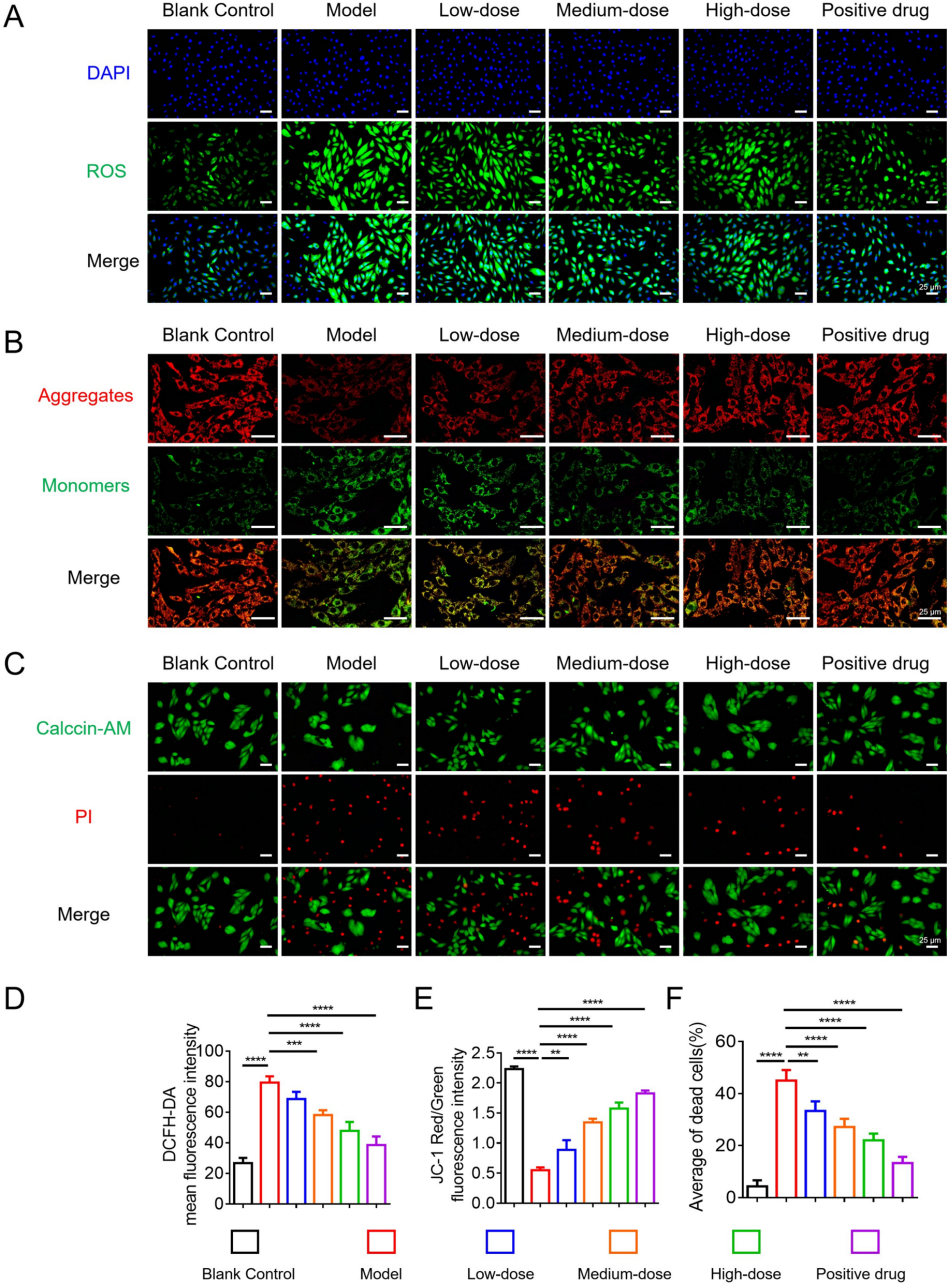
Effects of HYQT on ROS, mitochondrial membrane potential, and cell viability in H9C2 cell. **A** Staining for ROS detection (DAPI for nuclear staining, green for ROS; scale bar = 25 μm). **B** JC-1 staining to assess mitochondrial membrane potential (red for aggregates, indicating high membrane potential; green for monomers, indicating low membrane potential; scale bar = 25 μm). **C** Live/dead cell staining (Calcein-AM for live cells, green; PI for dead cells, red; scale bar = 25 μm). **D** Quantitative analysis of mean DCFH-DA fluorescence intensity (a probe for ROS). **E** Semi-quantitative analysis of JC-1 Red/Green fluorescence intensity ratio (an indicator of mitochondrial membrane potential).** F** Average percentage of dead cells. Data are presented as mean ± SD (n = 3). ^*^P < 0.05, ^**^P < 0.01, ^***^P < 0.001, ^****^P < 0.0001

Intracellular ROS levels in H9C2 cells across experimental groups were assessed via fluorescence staining (Fig. 2A, D). The results indicated a significant increase in ROS levels in the model group, as evidenced by markedly enhanced green fluorescence intensity. In contrast, treatment with HYQT resulted in a reduction of ROS fluorescence intensity in all dosage groups, demonstrating a discernible dose-dependent trend. These findings suggest that HYQT effectively attenuates intracellular ROS accumulation.

#### Measurement of mitochondrial membrane potential

Mitochondrial membrane potential (ΔΨm) was evaluated using fluorescent staining (Fig. 2B, E). Cells in the control group exhibited prominent red fluorescent aggregates, indicating normal ΔΨm. In the model group, a substantial increase in green fluorescence was observed, accompanied by a significant decrease in the Red/Green ratio compared to the blank control (*P* < 0.0001), indicating severe ISO-induced mitochondrial depolarization. HYQT treatment resulted in a dose-dependent recovery of the Red/Green ratio, confirming its protective effect against mitochondrial impairment.

#### Assessment of cell viability

Cell viability was determined via fluorescence-based staining (Fig. 2C, F). The model group showed reduced green fluorescence and enhanced red fluorescence, indicating decreased cell viability and an increased proportion of dead cells. Compared to the model group, all HYQT-treated groups exhibited increased green fluorescence and reduced cell death, with the high-dose group showing the highest cell viability and the lowest ratio of dead cells.

In summary, HYQT significantly mitigated ISO-induced injury by reducing ROS levels, preserving mitochondrial membrane potential, and enhancing cell viability in a dose-dependent manner.

#### HYQT attenuates isoproterenol‑induced cardiac hypertrophy

**Fig. 3:**
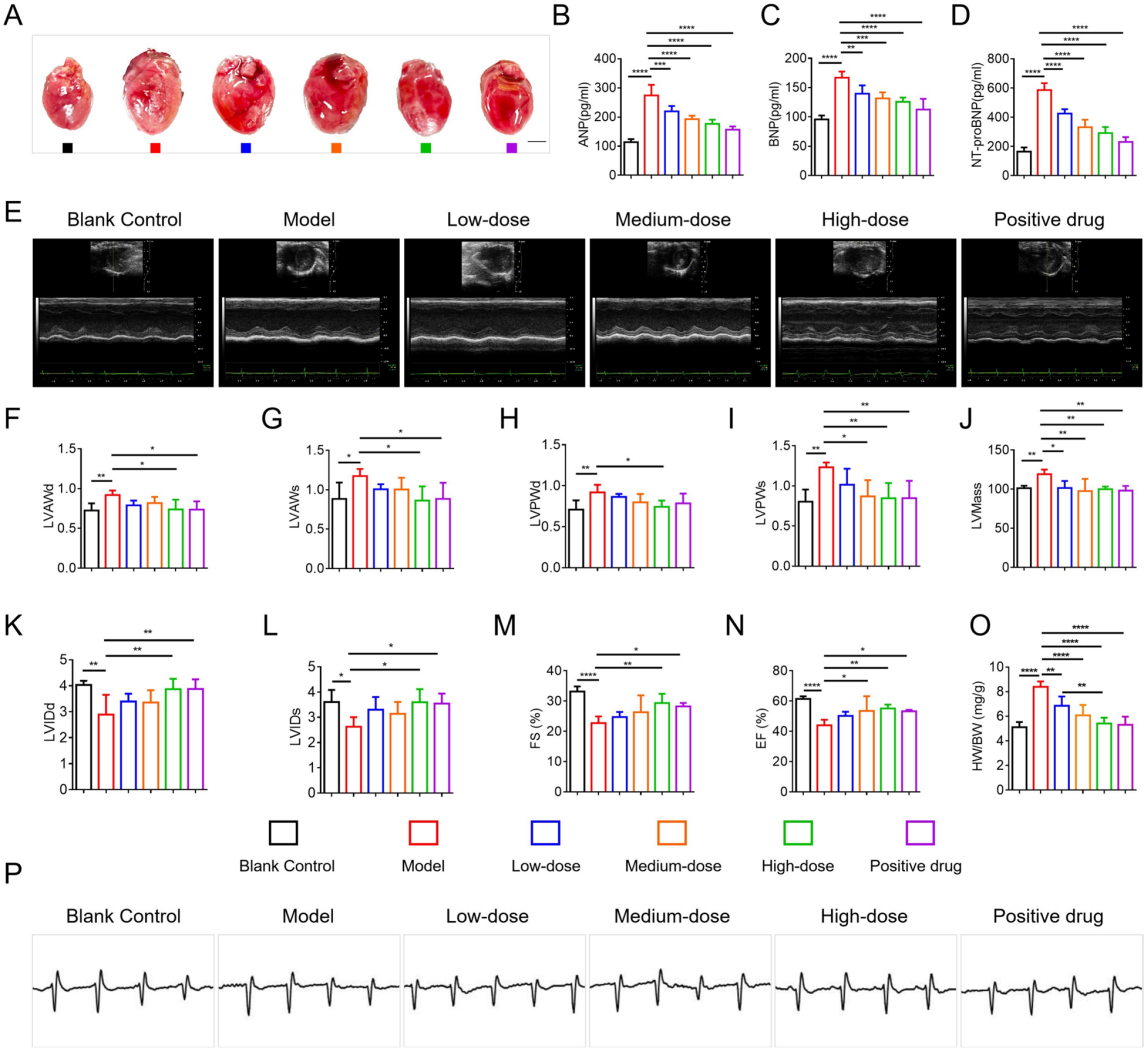
Effects of HYQT on cardiac morphology, function, and related molecular markers in a mouse model of hypertrophic cardiomyopathy. **A** Representative images of hearts from mice in different groups (scale bar: 2 mm). **B–D** Levels of ANP, BNP, and NT-proBNP in each group. **E** Representative M-mode echocardiographic images of LV structures in each group. **F** LV end-diastolic anterior wall thickness (LVAWd). **G** LV end-systolic anterior wall thickness (LVAWs). **H** LV end-diastolic posterior wall thickness (LVPWd).** I** LV end-systolic posterior wall thickness (LVPWs). **J** LV mass. **K** LV end-diastolic diameter (LVIDd). **L** LV end-systolic diameter (LVIDs). **M** fractional shortening (FS). **N** ejection fraction (EF). **O** heart weight-to-body weight ratio (HW/BW). **P** Representative electrocardiograms of mice in each group. Data are presented as mean ± SD. ^*^P < 0.05, ^**^P < 0.01, ^***^P < 0.001, ^****^P < 0.0001

To evaluate the therapeutic effect of HYQT on ISO‑induced cardiac enlargement in mice, thirty-six six-week-old male C57BL/6 mice (SPF-grade) were randomly allocated into six groups (n = 6 per group): Control, Model (ISO 5 mg/kg, subcutaneously once daily for 2 weeks), Low-dose HYQT, Medium-dose HYQT, High-dose HYQT, and Positive control (ISO + nicorandil). After 4 weeks of treatment, hearts were harvested. Bright-field microscopic images showed marked cardiac enlargement in the ISO group, which was dose-dependently attenuated by HYQT (Fig. 3A). ANP, BNP, and NT‑proBNP are well-established biomarkers of pathological cardiac hypertrophy [20]. In this study, ISO administration markedly increased expression levels of ANP, BNP, and NT‑proBNP in mouse myocardium. HYQT treatment dose-dependently reversed these elevations, with each of the low, medium, and high-dose groups showing significant differences versus the ISO model (*P* < 0.05) (Fig. 3B–D). These results indicate that HYQT effectively mitigates ISO-induced hypertrophic marker expression and inhibits cardiac hypertrophy progression.

#### Echocardiographic assessment of cardiac structure and function

Cardiac structure and function were evaluated by transthoracic echocardiography and heart weight to body weight ratio (HW/BW). Representative echocardiographic images are shown in Fig. 3E. Compared to the control group, mice in the ISO model group showed significant left-ventricular wall thickening and reduced LV cavity size, supporting successful induction of the HCM phenotype [21]. After four weeks of treatment, all HYQT groups (low, medium, high doses) and the nicorandil-treated positive control displayed reduced LV wall thickness and enlarged LV cavity dimensions. Among them, the medium- and high-dose HYQT groups and the nicorandil group exhibited the most pronounced improvements.

Quantitative cardiac structural parameters are summarized in Fig. 3F–J. In the ISO group, significant increases were observed in interventricular septal thickness (diastolic/systolic, IVSd/IVSs) and posterior wall thickness (LVPWd/LVPWs), along with elevated LV mass (LVMass), indicating pronounced structural remodeling [22]. All HYQT doses and nicorandil significantly reduced these parameters, with the high-dose group demonstrating reductions comparable to those seen with nicorandil treatment.

Functional indicators—LV internal diameter at end-diastole and end-systole (LVIDd/LVIDs), ejection fraction (EF), and fractional shortening (FS)—are depicted in Fig. 3K–O. The ISO group showed reductions in LVIDd and LVIDs, as well as significantly lowered EF and FS, indicative of impaired systolic function [23]. HYQT treatment dose-dependently restored these metrics, with medium- and high-dose cohorts, as well as the nicorandil group, showing the most significant recovery.

Furthermore, the HW/BW ratio was significantly elevated in the ISO group but was reduced in all HYQT-treated groups and the nicorandil-treated mice; the high-dose HYQT group showed the largest reduction. These findings demonstrate a dose-dependent effect of HYQT in preserving cardiac structure and function.

#### Electrocardiogram (ECG) analysis

Representative ECG tracings are presented in Fig. 3P. The control group exhibited normal sinus rhythm with physiologically unremarkable P wave, QRS complex, and T wave morphology. In contrast, the ISO model group displayed prolongation of the QRS duration, decreased QRS amplitude, and flattened or inverted T waves, consistent with myocardial hypertrophy and conduction–repolarization abnormalities [24–26]. HYQT intervention progressively improved ECG parameters. In the medium- and high-dose HYQT groups and in the nicorandil group, there was significant QRS narrowing, recovery of amplitude, and normalization of T-wave morphology. The high-dose HYQT group exhibited ECG patterns most closely resembling the controls, indicating effective restoration of myocardial electrophysiological integrity and repolarization.

These results demonstrate that HYQT significantly ameliorated cardiac structural and functional impairments in ISO-induced HCM, with efficacy comparable to that of nicorandil. Dose-dependent improvements in echocardiographic parameters—including ventricular wall thickness, left ventricular mass, ejection fraction, fractional shortening, left ventricular internal dimensions, and the heart weight-to-body weight ratio—along with normalization of electrocardiographic findings, indicate that HYQT effectively reversed ISO-induced hypertrophic remodeling and attenuated electrophysiological disturbances, likely through modulation of myocardial signaling pathways and preservation of structural integrity.

#### Histopathological assessment and anti-fibrotic effects of HYQT

**Fig. 4:**
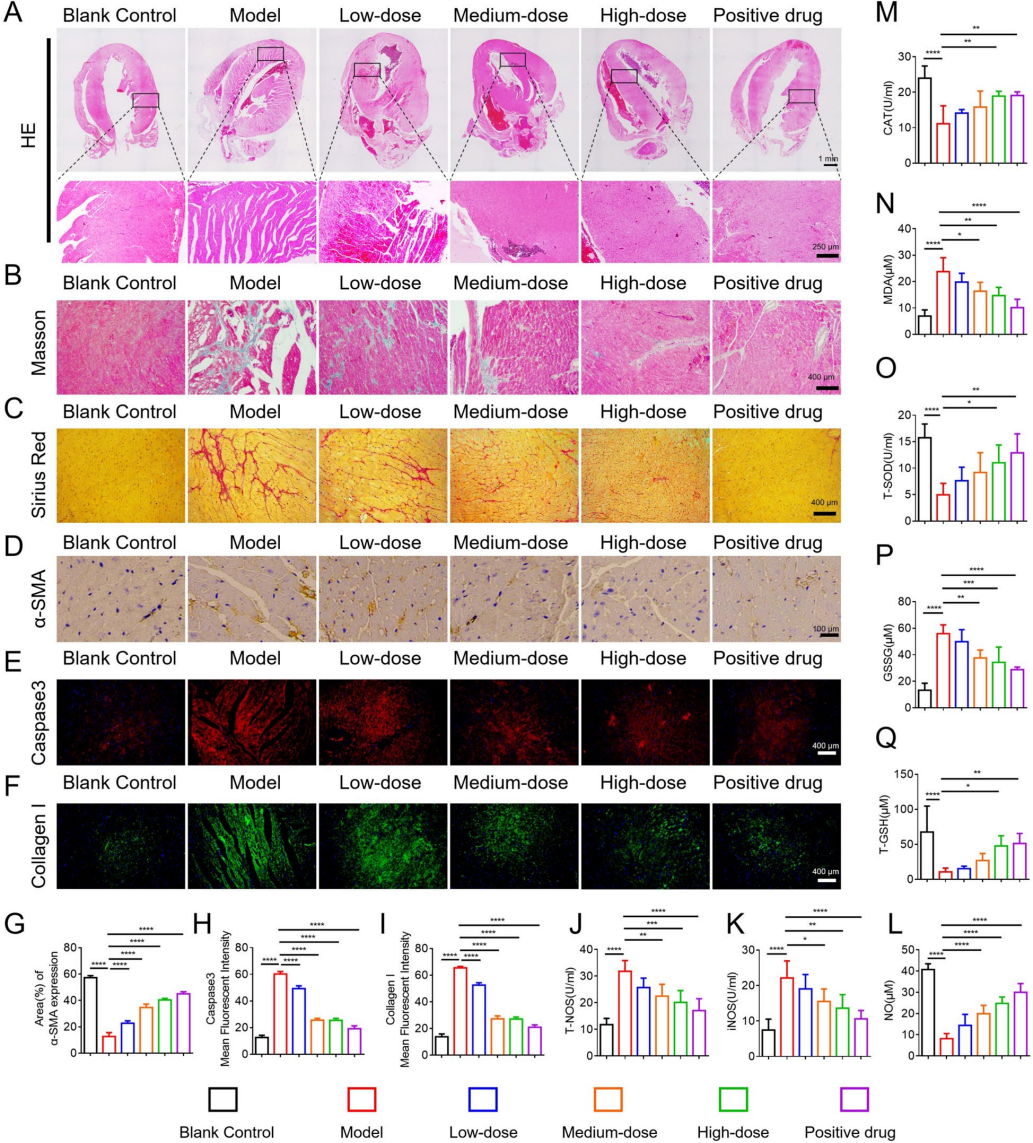
Effects of HYQT on myocardial histopathology, fibrosis, oxidative stress, and nitric oxide synthase-related indicators in a mouse model of HCM. **A** HE staining of myocardial tissue (upper panel: scale bar = 1 mm; lower panel: scale bar = 250 μm). **B** Masson’s staining for collagen deposition (scale bar = 400 μm). **C** Sirius Red staining for collagen fibers (scale bar = 400 μm). **D** Immunohistochemical staining of α-SMA (scale bar = 100 μm). **E** Immunofluorescence staining of Caspase-3 (scale bar = 400 μm). **F** Immunofluorescence staining of Collagen I (scale bar = 400 μm). **G** Quantitative analysis of α-SMA-positive area. **H** Mean fluorescence intensity of Caspase-3. **I** Mean fluorescence intensity of Collagen I. **J** Activity of T-NOS. **K** Activity of iNOS. **L** Content of NO. **M** Activity of CAT. **N** MDA level. **O** Activity of T-SOD. **P** GSSG level. **Q** T-GSH level. Data are presented as mean ± SD. ^*^P < 0.05, ^**^P < 0.01, ^***^P < 0.001, ^****^P < 0.0001

Hematoxylin and Eosin (HE) staining (Fig. 4A) revealed that, compared to the control group, ISO-treated mice exhibited marked disorganization of myocardial fibers, pronounced edema, vascular congestion, and substantial infiltration of inflammatory cells. Treatment with HYQT notably improved myocardial fiber alignment and significantly reduced inflammatory cell infiltration, indicating a mitigation of myocardial tissue damage.

Masson’s Trichrome and Sirius Red staining (Fig. 4B, C) further highlighted cardiac fibrosis and collagen deposition as key pathological features of HCM. Masson’s trichrome staining showed extensive interstitial fibrosis with dense blue-stained collagen in the ISO group. HYQT administration reduced collagen deposition in a dose-dependent manner, with the high-dose HYQT group demonstrating a degree of fibrosis attenuation comparable to that observed with nicorandil, a known cardioprotective agent. Sirius Red staining supported these findings: ISO-treated hearts displayed increased red collagen fiber density, while escalating doses of HYQT progressively diminished collagen content, reinforcing its anti-fibrotic potential.

IHC/IF analyses of α‑SMA and Collagen I (Fig. 4D–F) showed that α‑SMA, a marker of myofibroblast activation, was scarcely expressed in control myocardium but was significantly upregulated following ISO treatment. HYQT suppressed α-SMA expression in a dose-dependent manner. In line with this, immunofluorescence staining of Collagen I showed heavy ECM deposition in ISO-treated hearts, which was markedly reduced by high-dose HYQT, restoring collagen expression toward levels observed in the control group. These results emphasize HYQT’s efficacy in attenuating ECM accumulation. Caspase‑3 immunofluorescence staining (Fig. 4E) showed strong red fluorescence signals in the myocardium of ISO-treated mice, indicative of elevated cardiomyocyte apoptosis. HYQT treatment significantly reduced Caspase‑3 expression in a dose-dependent manner, suggesting that its cardioprotective effect includes inhibition of apoptosis in addition to fibrosis attenuation.

To assess the impact of HYQT on oxidative stress in the HCM mouse model, key indicators were measured, including total nitric oxide synthase (T-NOS) and inducible nitric oxide synthase (iNOS) activities (Fig. 4J–K). In the ISO model group, oxidative stress and inflammation were significantly increased, leading to elevated T-NOS activity (*P* < 0.01) and markedly upregulated iNOS activity (*P* < 0.001), reflecting an imbalance in nitric oxide signaling. Following HYQT intervention, T-NOS activity decreased in a dose-dependent manner across low-, medium-, and high-dose groups, with the high-dose group showing no significant difference from the control group. iNOS activity was significantly downregulated in a dose-dependent manner (*P* < 0.01), suggesting that HYQT can suppress excessive iNOS activation and restore nitric oxide signaling balance.

Malondialdehyde (MDA) and total superoxide dismutase (T-SOD) activities were also assessed (Fig. 4N, O). The model group exhibited a significant increase in MDA levels (*P* < 0.001) and a significant decrease in T-SOD activity (*P* < 0.01), reflecting severe myocardial lipid peroxidation damage. After HYQT treatment, MDA levels decreased in a dose-dependent manner (*P* < 0.05 or *P* < 0.01), with the high-dose group approaching levels seen in the control group. T-SOD activity significantly increased in the medium- and high-dose groups (*P* < 0.05), indicating that HYQT enhances myocardial antioxidant enzyme activity and alleviates oxidative damage. Additionally, catalase (CAT), oxidized glutathione (GSSG), and total glutathione (T-GSH) levels were measured (Fig. 4M, P, Q). The model group showed a significant decrease in CAT activity (*P* < 0.01), an increase in GSSG levels (*P* < 0.05), and a decrease in T-GSH levels (*P* < 0.01), suggesting impairment of the myocardial antioxidant defense system, particularly the glutathione redox cycle. HYQT treatment resulted in a significant recovery of CAT activity in the high-dose group (*P* < 0.05), a decrease in GSSG levels (*P* < 0.05), and an increase in T-GSH levels (*P* < 0.01), confirming that HYQT can repair myocardial antioxidant capacity and restore redox balance in HCM mice.

ISO-induced HCM is a well-established preclinical model characterized by β-adrenergic overstimulation, oxidative stress, and fibrotic remodeling [27]. Previous studies have shown that nicorandil can prevent ISO-induced cardiac fibrosis and suppress ROS generation [28]. In this context, HYQT exhibited robust protective effects against ISO-induced structural disruption and apoptosis, underscoring its therapeutic potential in HCM.

#### HYQT Improves myocardial perfusion, angiogenesis, and inflammation in HCM mice

**Fig. 5:**
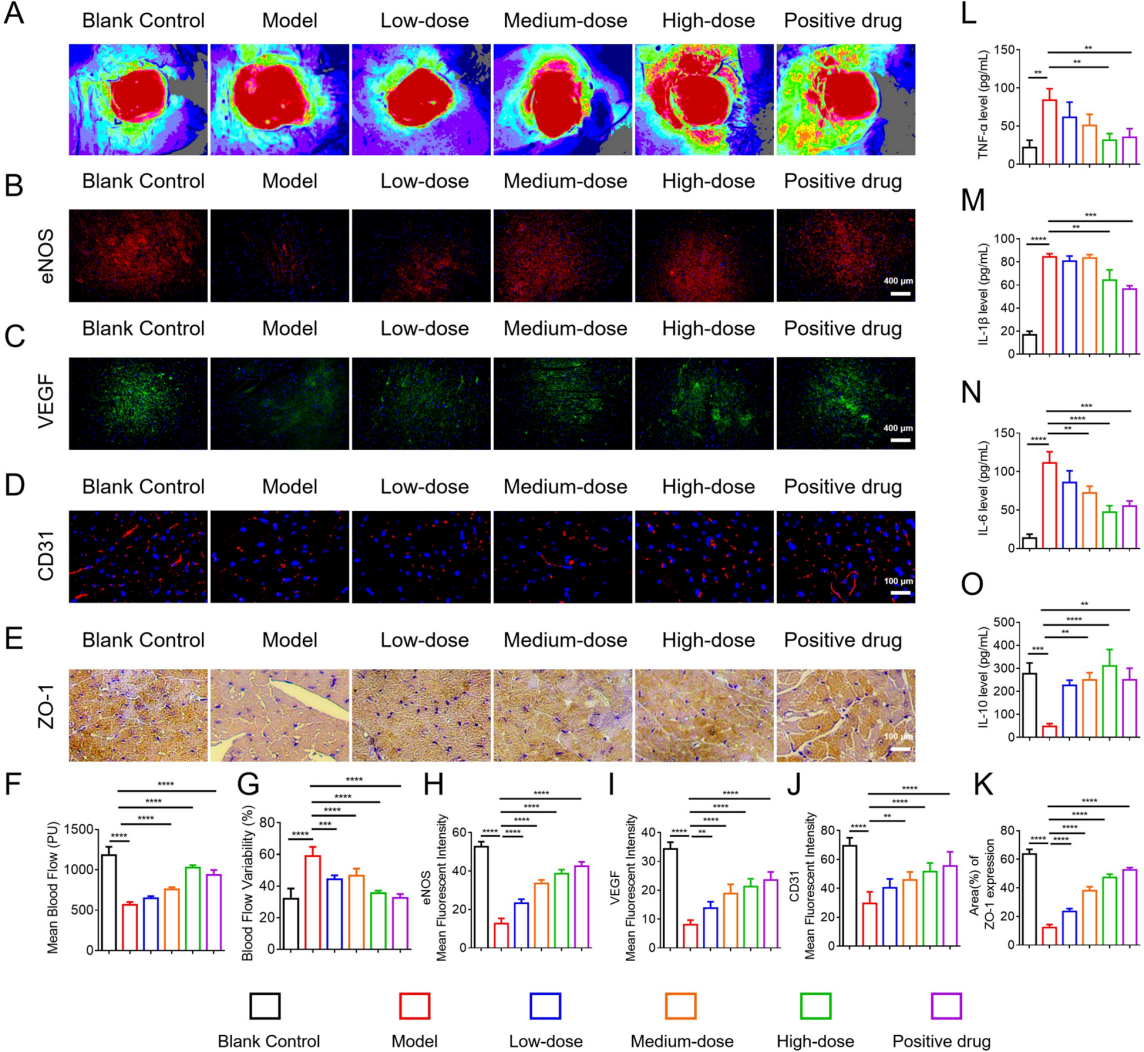
Effects of HYQT on myocardial blood flow, angiogenesis-related markers, and inflammatory factors in a mouse model of HCM. **A** Representative laser speckle images of myocardial blood flow distribution in each group. **B** Immunofluorescence staining of eNOS (scale bar = 400 μm). **C** Immunofluorescence staining of VEGF (scale bar = 400 μm). **D** Immunofluorescence staining of CD31 (scale bar = 100 μm). **E** Immunohistochemical staining of ZO-1 (scale bar = 100 μm). **F** Quantitative analysis of mean myocardial blood flow. **G** Blood flow variability analysis. **H** Mean fluorescent intensity of eNOS. **I** Mean fluorescent intensity of VEGF. **J** Mean fluorescent intensity of CD31. **K** Positive area percentage of ZO-1. **L–O** Levels of inflammatory factors: **L** TNF-α, **M** IL-1β, **N** IL-6, **O** IL-10. Data are presented as mean ± SD. ^*^P < 0.05, ^**^P < 0.01, ^***^P < 0.001, ^****^P < 0.0001

Myocardial blood flow perfusion (Fig. 5A, F, G) assessed by laser speckle contrast imaging demonstrated uniform perfusion in the control group. In contrast, ISO-treated mice exhibited significantly reduced myocardial blood flow (Mean Blood Flow) (*P* < 0.0001) and markedly increased perfusion heterogeneity (Blood Flow Availability) (*P* < 0.0001), indicative of microvascular dysfunction associated with HCM. HYQT treatment restored perfusion in a dose-dependent manner: Mean Blood Flow was significantly increased in the low-, medium-, and high-dose HYQT groups (*P* < 0.05 or *P* < 0.01), with the high-dose group nearly reaching control levels. Concurrently, Blood Flow Availability decreased significantly at all dose levels (*P* < 0.0001), reflecting improved homogeneity of myocardial perfusion. These findings suggest that HYQT effectively alleviates microvascular impairment in HCM. This application of laser speckle imaging aligns with prior studies utilizing the technique to detect microvascular alterations [29].

Angiogenesis-related markers—eNOS, NO, VEGF, and CD31 (Fig. 5B–D, H–J)—were evaluated to explore the mechanisms underlying improved perfusion. ISO treatment significantly downregulated endothelial nitric oxide synthase (eNOS) expression (*P* < 0.0001), indicating endothelial dysfunction. HYQT treatment dose-dependently increased eNOS fluorescence intensity (*P* < 0.0001), comparable to the effect observed with nicorandil, suggesting restoration of endothelial-dependent vasodilation. Similarly, nitric oxide (NO) levels, which were significantly diminished in ISO-treated mice (*P* < 0.01), increased following HYQT administration in a dose-dependent fashion (*P* < 0.05) (Fig. 5L). VEGF expression was also markedly suppressed in the ISO group (*P* < 0.0001) but significantly upregulated by HYQT at all tested doses (*P* < 0.05 or *P* < 0.01), with high-dose HYQT restoring VEGF levels to those of the control group. CD31-positive microvessel density was significantly reduced in ISO mice (*P* < 0.0001), indicating impaired angiogenesis. HYQT treatment enhanced CD31 expression, especially in the medium- and high-dose groups (*P* < 0.0001), confirming its pro-angiogenic potential. These results are consistent with previous reports showing that nicorandil enhances microvascular density and upregulates eNOS and VEGF in hypertensive cardiac hypertrophy models [30].

Vascular integrity and inflammatory microenvironment (Fig. 5E, K; L–O) were also evaluated. ZO-1 immunostaining revealed continuous endothelial tight junctions in control mice, which appeared disrupted in ISO-treated mice (*P* < 0.0001), suggesting vascular barrier dysfunction. HYQT treatment, particularly at medium and high doses, significantly restored ZO-1 expression (*P* < 0.0001), indicating improved endothelial integrity. Furthermore, ISO treatment led to an imbalanced inflammatory state, with elevated levels of pro-inflammatory cytokines (TNF-α, IL-1β, IL-6) and reduced levels of the anti-inflammatory cytokine IL-10 (*P* < 0.0001). HYQT dose-dependently suppressed pro-inflammatory cytokine expression (*P* < 0.05 or *P* < 0.01) while restoring IL-10 levels (*P* < 0.05). Notably, high-dose HYQT normalized all measured cytokines to near-control levels, demonstrating its capacity to restore immune balance and modulate the inflammatory microenvironment in the hearts of HCM mice.

Collectively, HYQT enhances myocardial perfusion, promotes angiogenesis, strengthens endothelial integrity, and rebalances cardiac inflammation in HCM mice, with dose-dependent effects rivaling those of nicorandil. These outcomes highlight HYQT’s multifaceted vascular and immunomodulatory benefits in HCM models.

#### Identification of blood‑absorbed components of HYQT

**Fig. 6:**
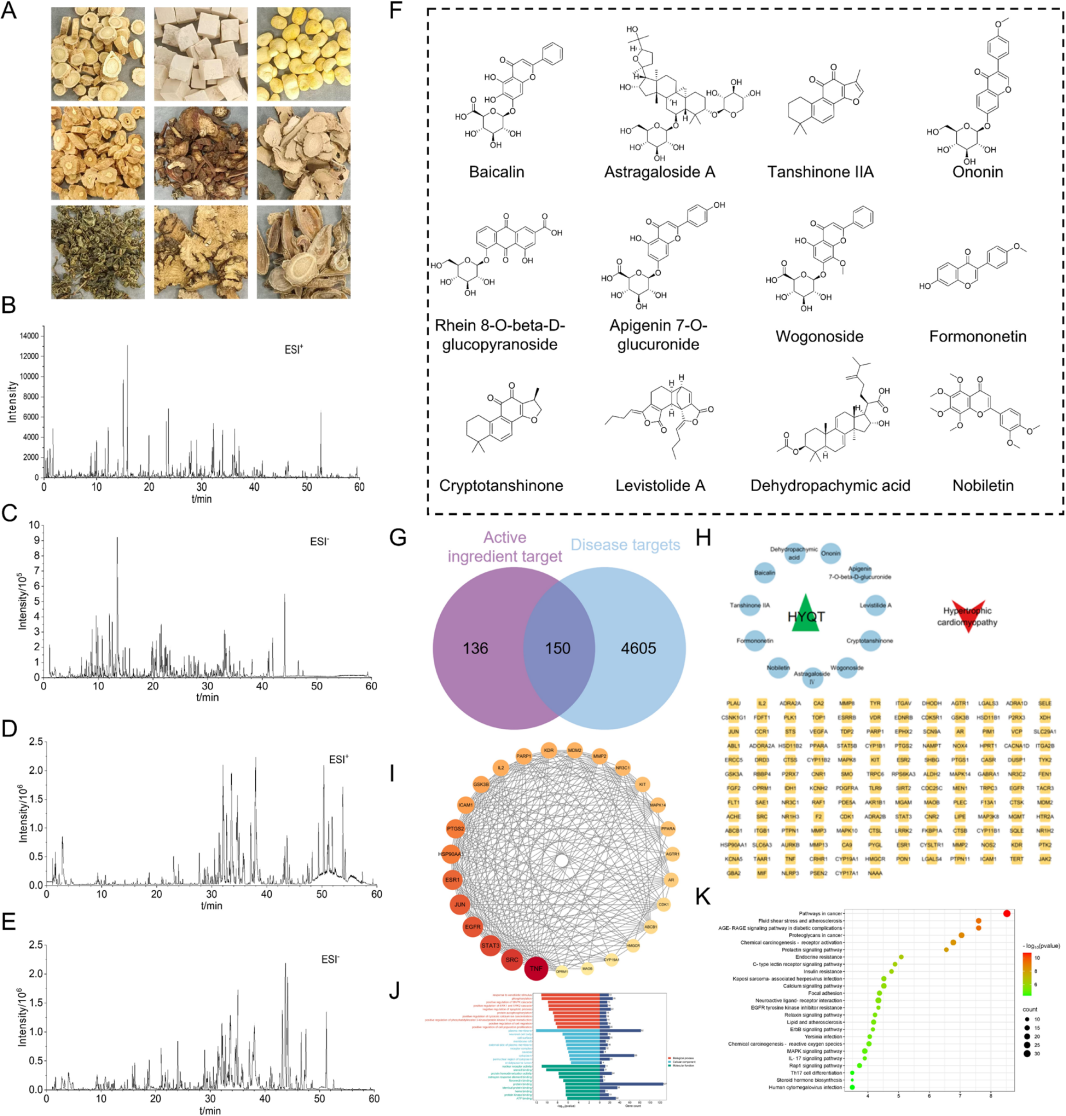
Chemical composition analysis and network pharmacology prediction of HYQT in the treatment of HCM. **A** Digital photographs of the medicinal herbs composing HYQT. **B** Base peak ion chromatogram (BPC) of HYQT in positive ion mode. **C** BPC of HYQT in negative ion mode. **D** BPC of serum containing HYQT in positive ion mode. **E** BPC of serum containing HYQT in negative ion mode. **F** Chemical structures of bioactive components absorbed into the bloodstream, including Baicalin, Astragaloside IV, and Tanshinone IIA, which represent the pharmacologically active substances exposed in vivo. **G** Venn diagram showing overlapping targets between HYQT-derived bioactive compounds (purple circle) and HCM-related disease targets (blue circle). **H** Compound–target–disease network constructed using Cytoscape 3.9.1. Triangles represent HYQT, circles represent bioactive compounds, inverted triangles represent the disease, and squares represent the overlapping targets. **I** Protein–protein interaction (PPI) network of core therapeutic targets. **J** Gene Ontology (GO) enrichment analysis of the potential therapeutic targets of HYQT for HCM. **K** KEGG pathway enrichment analysis (bubble plot) of the identified targets involved in HYQT’s treatment of HCM

To elucidate the material basis and molecular mechanisms underlying HYQT’s therapeutic effect on HCM, UHPLC‑Q‑TOF MS was employed to comprehensively analyze its chemical profile. Total ion chromatograms in both positive and negative ion modes exhibited multiple distinct peaks corresponding to HYQT constituents and blood‑absorbed metabolites (Fig. 6B–E). Using spectral matching—including compound MS/MS fragmentation, literature comparison, and reference standards—a total of 119 compounds were tentatively identified (Table S1), detailing chemical formulas, retention times, adduct forms, m/z values, mass errors, and fragment ions. Among these, 31 compounds were confirmed in plasma after oral administration (Table 2), including flavonoids (e.g., baicalin, wogonoside, apigenin 7-O‑β‑d‑glucuronide), anthraquinones (e.g., rhein 8‑O‑β‑d‑glucopyranoside), triterpenoid saponins (e.g., astragaloside IV), tanshinones (e.g., cryptotanshinone, tanshinone IIA), alkaloids (reserpine), amino acids, organic acids, bile acids, and vitamins. The molecular structures of a portion of the compounds detected in plasma are illustrated in Fig. 6F.

**Table 2:** Initial identification of compounds into the blood

No	Component name	Ion mode	Retention time (min)	Area	Formula	Precursor mass	Found At mass	Mass error (ppm)	Secondary fragment ions
1	Baicalin	–	9.85	1.29E + 06	C_21_H_18_O_11_	445.078	445.0773	− 0.7	269.0460、113.0247、85.0290
2	Rhein 8-O-beta-d-glucopyranoside	–	9.85	1.29E + 06	C_21_H_18_O_11_	445.078	445.0773	− 0.7	343.0170、271.0602
3	Apigenin 7-O-beta-d-glucuronide	–	9.85	1.29E + 06	C_21_H_18_O_11_	445.078	445.0773	− 0.7	269.0460、113.0247、85.0290
4	Wogonoside	–	9.85	4.00E + 05	C_22_H_20_O_11_	459.093	460.101	− 0.2	459.0928、283.0613、268.0379、239.0356、211.0398、175.0247、113.0241、85.0293
5	Ononin	+	13.37	1.84E + 04	C_22_H_22_O_9_	431.134	431.1339	0.5	269.0816、213.0891
6	Formononetin	+	19.91	6.30E + 04	C_16_H_12_O_4_	269.081	269.0806	− 1.2	254.0569、237.0545、197.0597、181.0646
7	Astragaloside IV + HCOOH	–	21.5	2.82E + 04	C_41_H_68_O_14_ HCOOH	829.459	829.4603	1.4	783.4518
8	Nobiletin	+	23.06	1.58E + 04	C_21_H_22_O_8_	403.139	403.1388	0.2	388.1163、373.0930、358.0683、185.0789
9	Cryptotanshinone	+	32.1	9.00E + 04	C_19_H_20_O_3_	297.149	297.1483	− 0.1	282.1244、254.0932、209.0959
10	Tanshinone IIA	+	36.6	2.10E + 05	C_19_H_18_O_3_	295.133	295.1326	− 0.5	249.0909、234.0677、178.0779
11	Levistilide A	+	37.2	1.50E + 05	C_24_H_28_O_4_	381.206	381.2058	− 2.1	191.1062、173.0962
12	DehydropachyMic acid	+	41.35	6.18E + 04	C_33_H_50_O_5_	527.373	527.3729	− 2.2	449.3410、353.2475、278.2024
13	Reserpine	+	59.29	3.59E + 05	C_33_H_40_N_2_O_9_	609.281	609.2804	− 0.4	448.1953、397.2124、195.0655、174.0912
14	4-Hydroxybenzaldehyde	+	1.07	1.23E + 06	C_7_H_6_O_2_	123.044	123.044	− 0.4	80.0494、78.0337、77.0386、53.0385
15	Leucine	+	1.25	7.95E + 04	C_6_H_13_NO_2_	132.102	132.1018	− 0.7	86.0963、69.0698、57.0574
16	Isoleucine	+	1.2	3.64E + 06	C_6_H_13_NO_2_	132.102	132.1017	− 1.2	86.0963、69.0698、57.0574
17	Thymine	+	1.56	1.71E + 05	C_5_H_6_N_2_O_2_	127.05	127.0501	− 1	110.0234、109.0388、84.0444、54.0339
18	Phenprobamate	+	1.61	8.89E + 06	C_9_H_11_NO_2_	166.086	166.086	− 1.5	120.0817、103.0539、91.0540、77.0383
19	Loganic acid	+	6.94	7.86E + 04	C_16_H_24_O_10_	377.144	377.1455	3.5	359.1313、243.0882、198.0674、172.0870、145.0772
20	Vitamin B2	+	6.94	8.05E + 04	C_17_H_20_N_4_O_6_	377.146	377.1457	0.4	359.1313、243.0882、198.0674、172.0870、145.0772
21	Paederoside	–	9.85	1.30E + 06	C_18_H_22_O_11_S	445.081	445.0779	− 7.1	269.0460、113.0247、85.0290
22	Glycocholic acid	–	21	2.37E + 07	C_26_H_43_NO_6_	464.302	464.3015	− 0.5	402.3013、74.0245
23	Glycyrrhizic acid	+	22.19	2.48E + 03	C_42_H_62_O_16_	823.411	823.4101	− 1.1	647.2773、453.3384、453.3536
24	Cholan-24-oic acid	–	24.06	3.31E + 07	C_24_H_40_O_5_	407.28	407.2801	− 0.4	343.2640、289.2173、251.2015
25	4'-Hydroxyacetophenone	+	24.37	1.14E + 04	C_8_H_8_O_2_	137.06	137.0597	0	95.0858、81.0697、79.0551、67.0543
26	Anisaldehyde	+	24.37	1.14E + 04	C_8_H_8_O_2_	137.06	137.0597	0	95.0858、81.0697、79.0551、67.0543
27	Glycoursodeoxycholic acid	–	24.69	5.09E + 06	C_26_H_43_NO_5_	448.307	448.307	0.3	386.3066、74.0246
28	Chenodeoxycholic acid	–	28.78	9.00E + 06	C_24_H_40_O_4_	391.285	391.2853	− 0.2	373.2737
29	Deoxycholic acid	–	28.78	9.00E + 06	C_24_H_40_O_4_	391.285	391.2853	− 0.2	373.2737
30	Ursodeoxycholic Acid	–	28.78	9.00E + 06	C_24_H_40_O_4_	391.285	391.2853	− 0.2	373.2737
31	Hyodeoxycholic acid	–	28.78	9.00E + 06	C_24_H_40_O_4_	391.285	391.2853	− 0.2	373.2737

The herb names are from The Pharmacopoeia of the People’s Republic of China (2020 Edition)

#### Network pharmacology prediction of HYQT targets in HCM

To investigate the potential molecular targets of HYQT in HCM, network pharmacology analyses were conducted. First, a search for “Hypertrophic cardiomyopathy” in the GeneCards, OMIM, and TTD databases yielded a combined total of 4755 disease-related targets. Next, SwissTargetPrediction and related tools were used to predict 150 putative protein targets of the 31 absorbed HYQT compounds (Fig. 6G).

Using Cytoscape 3.9.1, a “drug–compound–target–disease” network was constructed: triangles represent HYQT, circles represent active compounds, inverted triangles represent disease, and squares indicate overlapping targets (Fig. 6H). The 150 predicted targets overlapped with HCM-related targets; these overlapping proteins were input into STRING to build a protein–protein interaction (PPI) network. Non-interacting (isolated) nodes were excluded. The remaining network was analyzed in Cytoscape using the Network Analyzer plugin. Three topological metrics—betweenness centrality (BC), closeness centrality (CC), and degree—were calculated and compared to their respective median values. Targets exceeding the median across all three metrics were identified as core or hub proteins, with final ranking based on degree value (Fig. 6I).

#### Functional enrichment and pathway analysis

Gene Ontology (GO) enrichment analysis of the core targets identified: Biological Processes (BP, 492 terms)—including response to exogenous stimuli, regulation of phosphorylation, MAPK cascade activation (including ERK1/ERK2), negative regulation of apoptosis, and autophosphorylation. Cellular Components (CC, 71 terms)—notably plasma membrane, neuronal cell body, cell surface, lipid rafts, extracellular side of plasma membrane, and receptor complexes. Molecular Functions (MF, 114 terms)—such as nuclear receptor activity, steroid binding, protein homodimerization, estrogen response element binding, fibronectin binding, and protein binding (Fig. 6J).

KEGG pathway analysis revealed 120 significantly enriched signaling pathways related to HYQT’s action on HCM. The top 25 enriched pathways, visualized as a bubble chart (Fig. 6K), include cancer-related pathways, AGE-RAGE signaling in diabetic complications, prolactin signaling, C-type lectin receptor signaling, relaxin signaling, ErbB, MAPK, IL-17, and Rap1 signaling.

Among these, the MAPK signaling pathway stands out. Isoform-regulated MAPK cascades (ERK1/2, p38, JNK) mediate cellular responses to stress, proliferation, apoptosis, and inflammation; targeting these cascades has been shown to modulate cardiac hypertrophy and fibrosis [31]. The presence of multiple absorbed flavonoids (e.g. baicalin, apigenin) and tanshinones, which have been reported to modulate MAPK and inflammatory pathways, suggests that HYQT may act through these bioactive constituents [32]. The network model thus supports a multi-target, multi-pathway mechanism where HYQT’s bioactive compounds synergize to influence MAPK signaling, NO production, apoptosis, fibrosis, and inflammation in HCM. These analyses collectively provide a robust molecular and pharmacological rationale for HYQT’s therapeutic efficacy against ISO-induced hypertrophic cardiomyopathy.

#### Molecular docking simulation validates HYQT targets

Previous network pharmacology analysis identified the MAPK signaling pathway as a critical intervention target for HYQT in alleviating HCM. MAPK cascades—including ERK1/2, p38, and JNK—regulate oxidative stress, inflammation, apoptosis, and fibrosis in cardiovascular disease, and their overactivation accelerates myocardial fibrosis and microvascular dysfunction in HCM [15, 33, 34].

In addition to MAPK signaling, hypoxia-inducible factor HIF-1α was considered because of its pivotal role in oxygen metabolism, angiogenesis, and endothelial function. In HCM, downregulation of HIF-1α in the early stage impairs capillary formation and microvascular integrity. Importantly, aberrant MAPK activation and HIF-1α dysfunction often occur in parallel and may exacerbate each other through oxidative stress, fibrosis, and impaired microcirculation. Therefore, to comprehensively evaluate HYQT’s potential mechanisms, molecular docking simulations were performed against MAPK1, p38 MAPK, and HIF-1α. Ten representative active compounds were selected: dehydropachymic acid, baicalin, rhein 8-O‑β‑D‑glucopyranoside, apigenin 7-O‑β‑D‑glucuronide, ononin, formononetin, nobiletin, cryptotanshinone, tanshinone IIA, and levistilide A. All showed stable docking conformations with binding free energies below − 5 kcal/mol, indicating strong binding affinities.

**Fig. 7:**
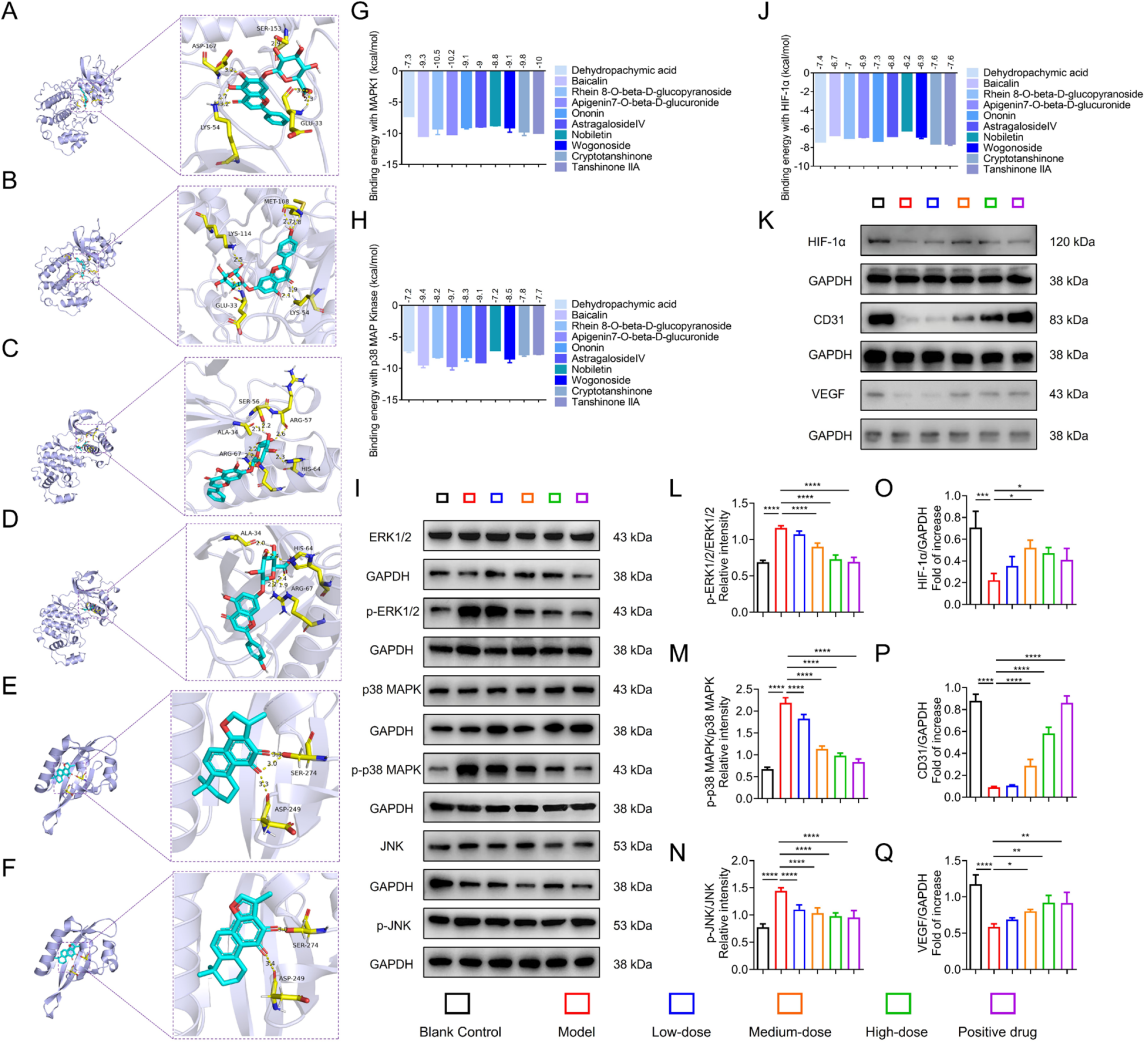
Molecular docking and validation of HYQT’s key components targeting MAPK signaling pathway and angiogenesis-related proteins in HCM. **A–F** Molecular docking visualization of core compounds with key proteins (MAPK1, p38, HIF-1α). Representative interaction diagrams of high—binding—affinity compounds: **A** MAPK1 vs. Baicalin, **B** MAPK1 vs. Apigenin-7-O-β-d-glucuronide, **C** p38 vs. Baicalein, **D** p38 vs. Apigenin-7-O-β-d-glucuronide, **E** HIF—1α vs. Cryptotanshinone, **F** HIF-1α vs. Tanshinone IIA. **G, H** Binding energy heatmaps of HYQT’s components with **G** p38 MAPK and **H** ERK1/2, where lower values indicate stronger binding affinity. **I** Western blot analysis of MAPK signaling pathway-related proteins: ERK1/2, p-ERK1/2, p38 MAPK, p-p38 MAPK, JNK, and p-JNK. GAPDH was used as a loading control. **J** Binding energy quantification of core compounds with core targets (HIF-1α). **K** Western blot analysis of angiogenesis-related proteins: HIF-1α, CD31, and VEGF. GAPDH was used as a loading control. **L–Q** Quantitative analysis of relative protein expression levels: **L** p-ERK1/2/ERK1/2, **M** p-p38 MAPK/p38 MAPK, **N** p-JNK/JNK, **O** HIF-1α/GAPDH, **P** CD31/GAPDH, **Q** VEGF/GAPDH. Data are presented as mean ± SD (n = 3). ^*^P < 0.05, ^**^P < 0.01, ^***^P < 0.001, ^****^P < 0.0001

Notably, baicalin (− 10.5 kcal/mol) and apigenin 7‑O‑β‑D‑glucuronide (− 10.2 kcal/mol) exhibited the strongest affinities for MAPK1, forming multiple hydrogen bonds at residues LYS-54, ASP-167, SER-153, and GLU-33 (Fig. 7A, B). Baicalin (− 9.4 kcal/mol) and the glucuronide (− 9.6 kcal/mol) also displayed the highest binding to p38 MAPK through interactions at HIS-64, ARG-67, and ALA-34 (Fig. 7C, D).

For HIF‑1α, cryptotanshinone and tanshinone IIA both showed binding energies of − 7.6 kcal/mol, forming hydrogen bonds with ASP-249 and SER-274 (Fig. 7E, F). These results suggest that baicalin and apigenin derivatives may serve as core bioactives in targeting MAPK branches, while tanshinone compounds may modulate HIF‑1α signaling.

These molecular docking results suggest a potential mechanism for HYQT’s multi-target action through the modulation of MAPK and HIF-1α signaling pathways. Baicalin and apigenin glucuronide likely represent key components mediating MAPK inhibition, whereas tanshinones may primarily affect HIF‑1α–regulated angiogenic pathways. This synergy underscores HYQT’s potential in orchestrating anti-fibrotic, antioxidant, and pro-angiogenic responses in HCM at the molecular level.

#### HYQT modulates the protein expression of MAPK and HIF-1α pathways

To validate the predictions from network pharmacology and molecular docking results, this study employed Western blot analysis to detect the expression levels of key proteins in the MAPK and HIF-1α signaling pathways in cardiac tissue. As shown in Fig. 7I and K, compared with the control group, the levels of phosphorylated ERK1/2 (p-ERK1/2), p-p38 MAPK, and p-JNK were significantly elevated in the model group, indicating aberrant activation of the MAPK pathway. In contrast, the expression of HIF-1α, VEGF, and CD31 was markedly reduced, suggesting potential impairment of the HIF-1α pathway. Despite upregulated MAPK signaling, the HIF-1α-mediated response may be compromised, failing to effectively activate angiogenesis-related genes, thereby contributing to decreased capillary density and microvascular dysfunction.

In the HYQT treatment groups, particularly the high-dose group, the expression levels of p-ERK1/2, p-p38 MAPK, and p-JNK were significantly decreased (*P* < 0.01 vs. model group), while the total levels of ERK1/2, p38, and JNK proteins showed no significant differences across groups. This indicates that HYQT inhibits the phosphorylation-dependent activation of the MAPK pathway (Fig. 7L–N). Concurrently, the protein expression of HIF-1α was significantly increased in the high-dose HYQT group (*P* < 0.01), with corresponding upregulation of its downstream target genes, VEGF and CD31 (*P* < 0.01), suggesting that HYQT enhances HIF-1α transcriptional activity and restores angiogenic capacity (Fig. 7O–Q).

In summary, HYQT may exert its therapeutic effects by modulating the MAPK and HIF-1α signaling pathways—suppressing pathological MAPK overactivation while potentially restoring HIF-1α-mediated angiogenic signaling—thereby improving myocardial hypertrophy, fibrosis, and microvascular dysfunction, suggesting its potential therapeutic value for HCM.

It is noteworthy that, although activation of the MAPK pathway is commonly observed in HCM, the stability and transcriptional regulatory capacity of HIF-1α may be compromised under pathological conditions. Factors such as chronic hypoxia, oxidative stress, and myocardial fibrosis may underlie this impairment. This impairment likely contributes to reduced expression of downstream targets VEGF and CD31 and restricted angiogenesis. Therefore, combined targeting of both MAPK and HIF-1α pathways, or specifically restoring the expression of HIF-1α downstream genes, may represent a critical therapeutic strategy for interrupting the vicious cycle of “ischemia–hypertrophy–inflammation–fibrosis” in HCM.

#### Safety evaluation of HYQT in mice and cellular assays

**Fig. 8:**
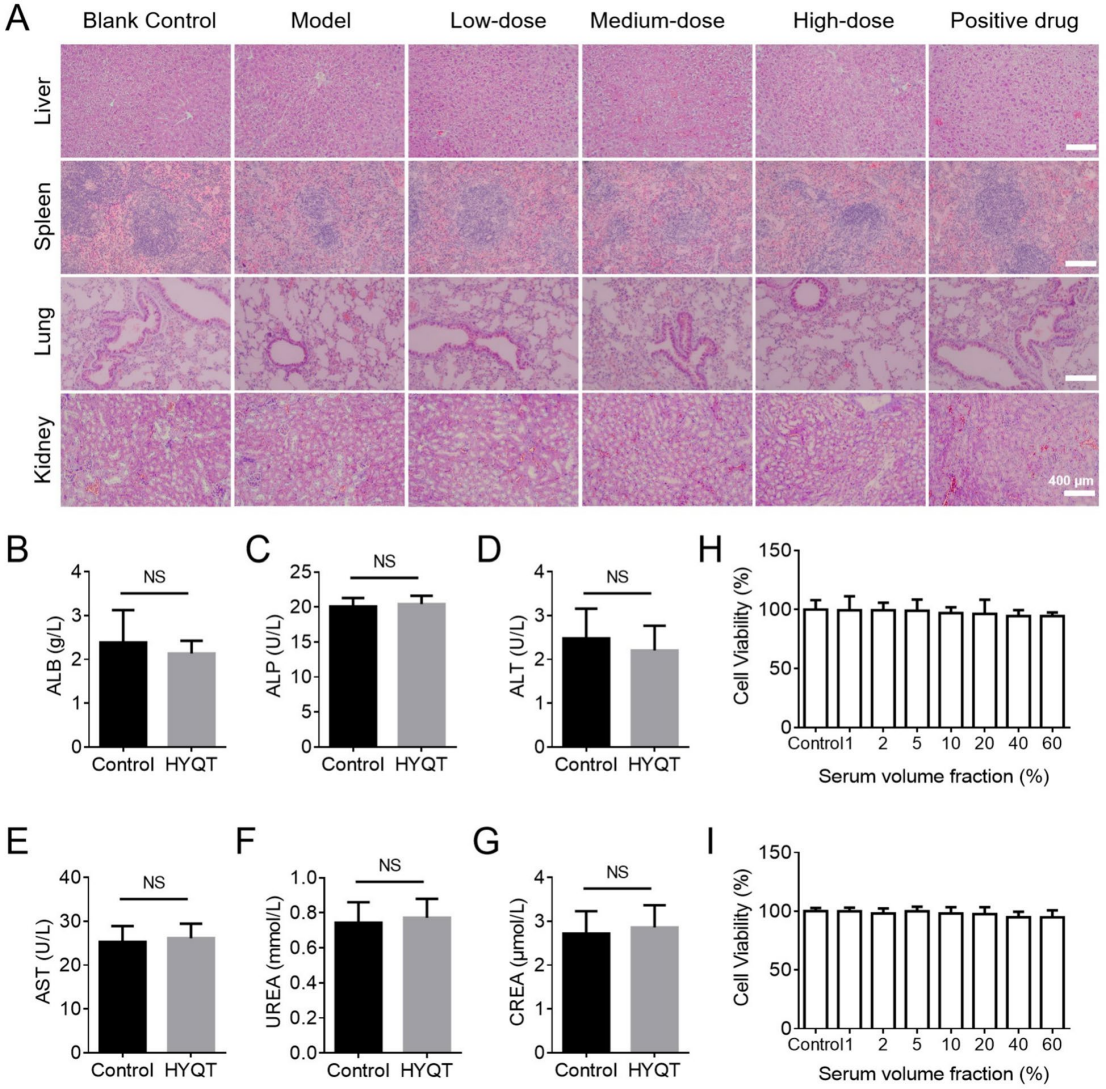
Safety evaluation of HYQT. **A** HE staining of major organs (liver, spleen, lung, kidney) from mice in different groups (scale bar = 400 μm). **B–G** Serum levels of liver and kidney function indicators in control and HYQT-treated groups: **B** ALB, **C** ALP, **D** ALT, **E** AST, **F** UREA, **G** CREA. “NS” indicates no significant difference (*P* > 0.05, unpaired t-test) (n = 3). **H** Cytotoxicity assessment of HYQT-containing serum on H9C2 Cell: Cell viability detected by CCK-8 assay in different serum volume fractions. **I** Cytotoxicity assessment of HYQT-containing serum on HCMECs (n = 5). Data are presented as mean ± SD

Histopathological examination of the liver, spleen, lungs, and kidneys revealed no evidence of toxicity or structural abnormalities in any HYQT-treated group, regardless of dose, indicating a favorable in vivo safety profile (Fig. 8A).

To further assess hepatic and renal safety, serum biochemical parameters were measured. Levels of albumin (ALB), alkaline phosphatase (ALP), alanine aminotransferase (ALT), and aspartate aminotransferase (AST) in HYQT-treated mice were comparable to those in the control group, indicating no hepatocellular damage or impairment of hepatic synthetic function (Fig. 8B–E). Similarly, blood urea nitrogen (UREA) and creatinine (CREA) levels showed no significant differences between groups, suggesting preserved renal function (Fig. 8F–G).

In vitro cytotoxicity assays demonstrated that HYQT-containing serum (1–60% v/v) did not adversely affect the viability of H9C2 cardiomyocytes or human cardiac microvascular endothelial cells (HCMECs), with cell viability exceeding 90% at all tested concentrations (Fig. 8H–I). These findings confirm that HYQT is non-cytotoxic to cardiac and endothelial cells within the effective dosage range.

Taken together, these results indicate that HYQT possesses a high safety margin, characterized by the absence of histopathological lesions in major organs, stable liver and kidney function markers, and negligible cytotoxicity in key cardiac-related cell types.

#### In vitro functional validation of HYQT on MAPK and HIF‑1α pathway

**Fig. 9:**
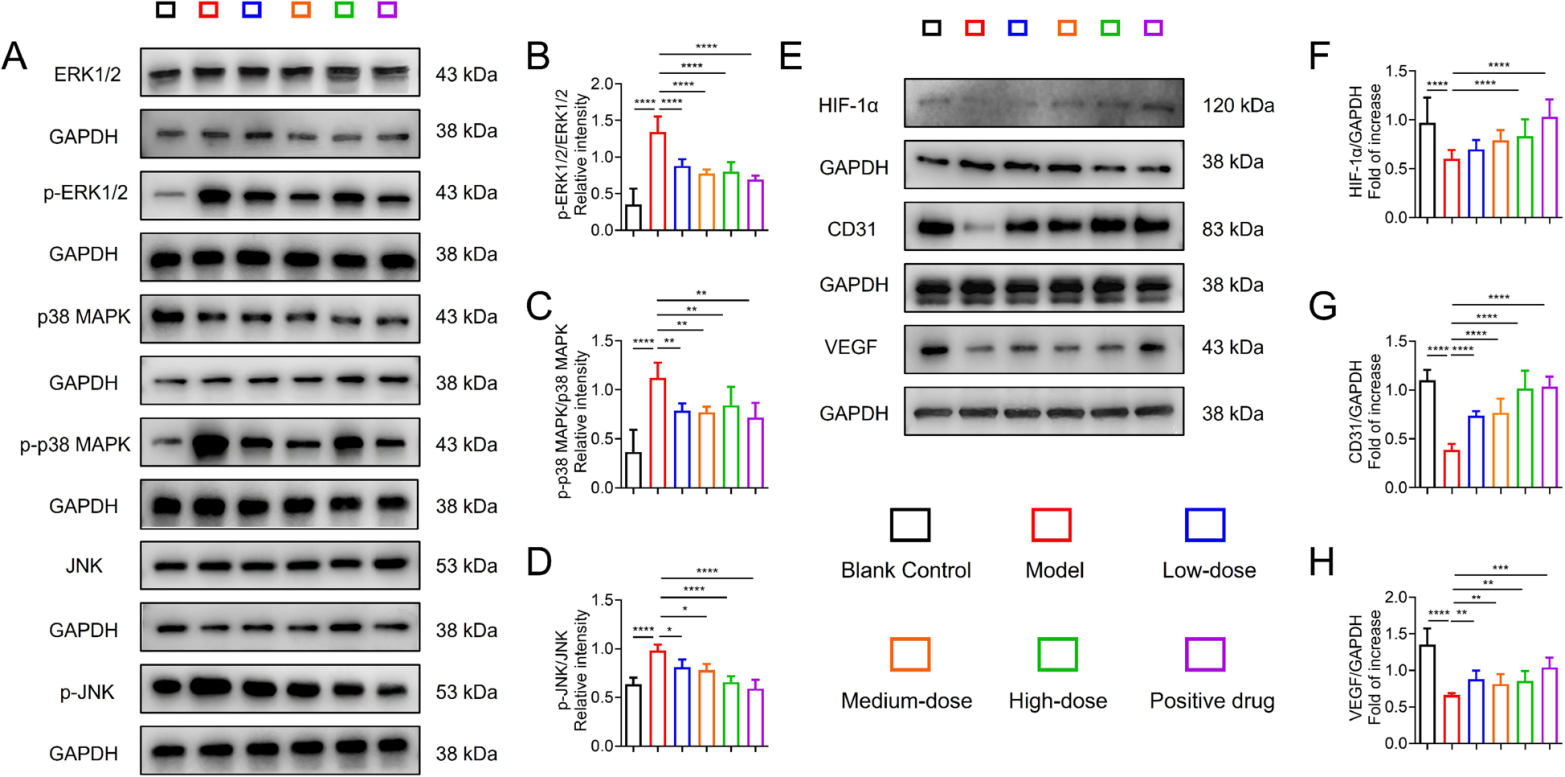
In vitro validation of HYQT regulating MAPK and HIF-1α pathway in ISO-induced cell injury models. **A** Western blot analysis of MAPK pathway-related proteins (ERK1/2, p-ERK1/2, p38 MAPK, p-p38 MAPK, JNK, p-JNK) in H9C2 cells. GAPDH served as a loading control. **B–D** Quantitative analysis of relative phosphorylation levels: **B** p-ERK1/2/ERK1/2, **C** p-p38 MAPK/p38 MAPK, **D** p-JNK/JNK. **E** Western blot analysis of HIF-1α pathway-related proteins (HIF-1α, VEGF, CD31) in HCMECs. GAPDH was used as a loading control. **F–H** Quantitative analysis of relative protein expression levels: **F** HIF-1α/GAPDH, **G** CD31/GAPDH, **H** VEGF/GAPDH. Data are presented as mean ± SD (n = 3). ^*^P < 0.05, ^**^P < 0.01, ^***^P < 0.001, ^****^P < 0.0001

To directly evaluate the effects of HYQT on HCM, an ISO–induced injury model was established in H9C2 cells and treated with different concentrations of HYQT (Fig. 9A–D). Compared with the blank control group, ISO stimulation markedly increased the phosphorylation levels of key MAPK signaling components, including ERK1/2, p38, and JNK. HYQT treatment effectively reversed these changes and significantly suppressed aberrant MAPK phosphorylation, suggesting that inhibition of MAPK overactivation may underlie the anti-HCM effects of HYQT.

**Fig. 10:**
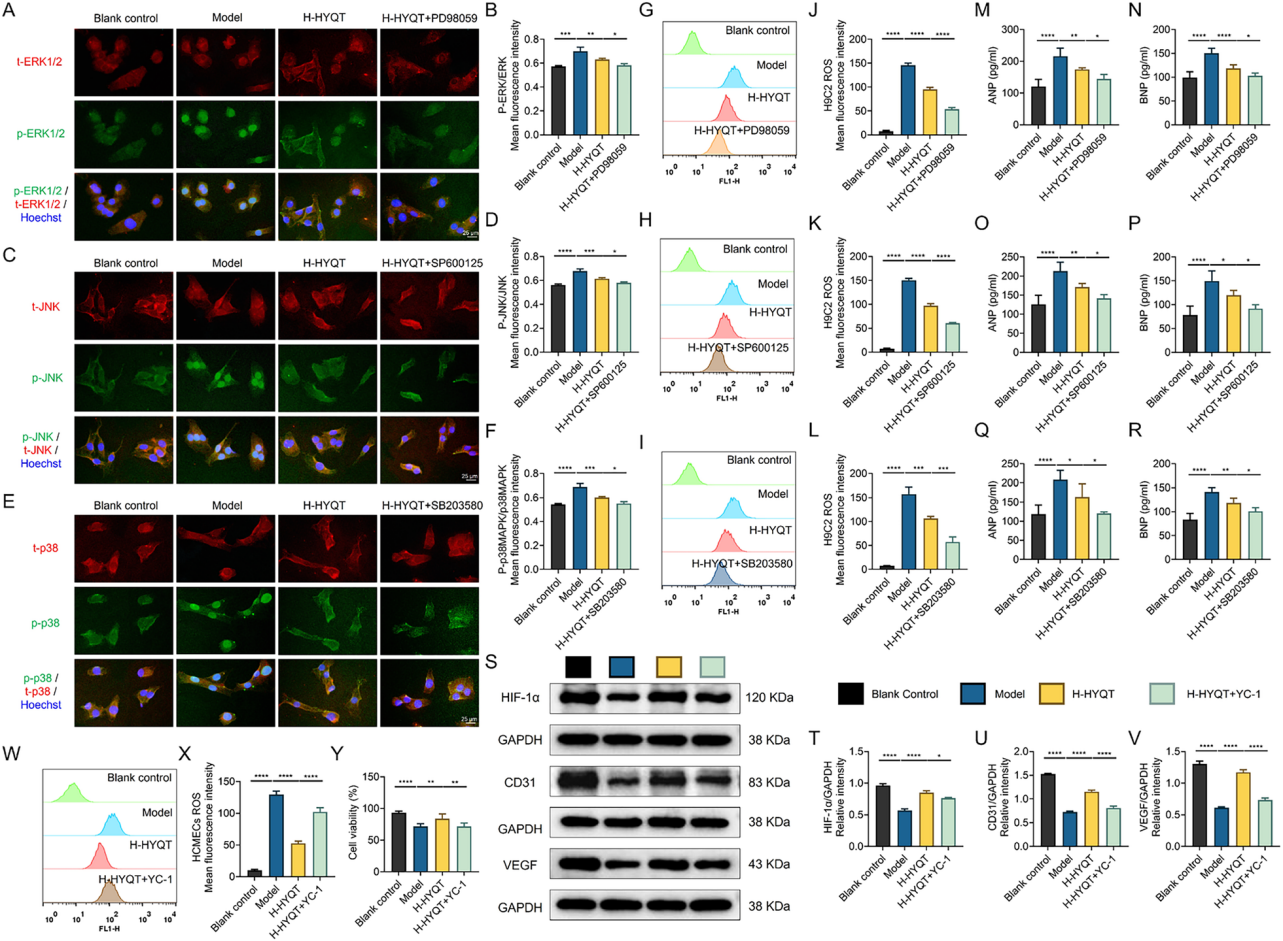
Pharmacological inhibition confirms the involvement of MAPK and HIF-1α signaling pathways in the protective effects of HYQT. **A, C, E** Immunofluorescence staining of MAPK signaling components in H9C2 cardiomyocytes. Representative images show total and phosphorylated ERK1/2 (**A**), JNK (**C**), and p38 (**E**). Nuclei were counterstained with Hoechst. Cells were divided into four groups: Blank control, Model, high-dose HYQT (H-HYQT), and H-HYQT combined with the corresponding pathway inhibitor (PD98059 for ERK, SP600125 for JNK, and SB203580 for p38). **B, D, F** Semi-quantitative analysis of immunofluorescence intensity, presented as the ratio of phosphorylated MAPK to total MAPK for ERK1/2 (**B**), JNK (**D**), and p38 (**F**). **G, H, I** Representative flow cytometry histograms showing intracellular reactive oxygen species (ROS) levels in H9C2 cells under the corresponding treatments for ERK (**G**), JNK (**H**), and p38 (**I**) pathway inhibition experiments. **J, K, L** Quantitative analysis of intracellular ROS levels in H9C2 cells corresponding to panels **G–I**. **M–R** Levels of hypertrophic markers ANP and BNP in H9C2 cells under the indicated treatments, corresponding to ERK (**M, N**), JNK (**O, P**), and p38 (**Q, R**) inhibition conditions. **S** Western blot analysis of HIF-1α, CD31, and VEGF expression in HCMECs cells treated with Blank control, Model, H-HYQT, or H-HYQT combined with the HIF-1α inhibitor YC-1. GAPDH was used as a loading control. **T–V** Semi-quantitative analysis of HIF-1α (**T**), CD31 (**U**), and VEGF (**V**) protein expression normalized to GAPDH in HCMECs. **W** Representative flow cytometry histograms of intracellular ROS levels in HCMECs under the indicated treatments. **X** Quantitative analysis of intracellular ROS levels corresponding to panel **W**. **Y** Cell viability of HCMECs assessed by CCK-8 assay under different treatment conditions. Data are presented as mean ± SD (n = 3). ^*^P < 0.05, ^**^P < 0.01, ^***^P < 0.001, ^****^P < 0.0001

To further determine whether MAPK signaling is functionally required for the cardioprotective effects of HYQT, pathway-specific pharmacological inhibition experiments were subsequently performed using the high dose of HYQT (H-HYQT) (Fig. 10A–R). In H9C2 cells, H-HYQT was co-administered with selective inhibitors targeting distinct MAPK branches, including PD98059 (ERK inhibitor), SP600125 (JNK inhibitor), and SB203580 (p38 inhibitor). Immunofluorescence analyses showed that, compared with H-HYQT treatment alone, combined inhibitor treatment further reduced the ratios of phosphorylated to total MAPK proteins (Fig. 10A–F). Functionally, inhibition of MAPK signaling led to a more pronounced attenuation of ISO-induced cellular injury, as evidenced by further reductions in intracellular ROS levels (Fig. 10G–L) and significantly decreased expression of HCM markers ANP and BNP (Fig. 10M–R). These results indicate that MAPK signaling represents a key regulatory node in ISO-induced cardiomyocyte injury and that the cardioprotective effects of HYQT are at least partially dependent on suppression of this pathway.

In parallel, to investigate the direct effects of HYQT on microvascular endothelial function, an ISO-induced injury model was established in HCMECs (Fig. 9E–H). ISO exposure significantly downregulated the expression of HIF-1α and its downstream angiogenic effectors, VEGF and CD31. Treatment with HYQT markedly restored the expression of HIF-1α, VEGF, and CD31, suggesting reactivation of HIF-1α–mediated angiogenic signaling under stress conditions.

To further validate the necessity of HIF-1α signaling in mediating the endothelial protective effects of HYQT, pharmacological inhibition experiments were likewise conducted using the high dose of HYQT, in combination with the well-established HIF-1α inhibitor YC-1 (Fig. 10S–Y). Western blot analyses demonstrated that YC-1 co-treatment markedly reversed the H-HYQT–induced upregulation of HIF-1α, VEGF, and CD31 (Fig. 10S–V). Functionally, inhibition of HIF-1α partially abolished the H-HYQT–mediated reduction in intracellular ROS levels (Fig. 10W, X) and attenuated its protective effects on endothelial cell viability (Fig. 10Y). These findings indicate that activation of HIF-1α signaling is required for the endothelial protective actions of HYQT under ISO-induced stress conditions.

Collectively, these data suggest that HYQT reestablishes microvascular homeostasis through dual mechanisms—by inhibiting aberrant MAPK activation and, in parallel, restoring HIF-1α-driven angiogenic signaling. This coordinated regulation effectively disrupts the “ischemia–hypertrophy–inflammation–fibrosis” vicious cycle in HCM and highlights HYQT as a promising therapeutic strategy.

## Discussion

This study examines the simultaneous regulatory effects of HYQT on cardiomyocytes and microvascular endothelial cells, providing new insights into the treatment of HCM. HCM is characterized by asymmetric myocardial hypertrophy, interstitial fibrosis and microvascular dysfunction, which contribute to impaired diastolic filling, increased arrhythmia risk, and reduced myocardial perfusion. These changes lead to complications such as heart failure and sudden cardiac death [35–37]. While conventional therapies often focus on individual molecular targets or pathways, our findings suggest that HYQT exerts simultaneous effects across cardiomyocytes and microvascular endothelial cells, reflecting the multi-target characteristics of Traditional Chinese Medicine (TCM).

Coronary microvascular dysfunction (CMD) plays a critical role in HCM. It manifests as reduced coronary flow reserve (CFR), impaired nitric oxide-mediated vasodilation and arteriolar wall thickening. Clinical imaging demonstrates that reduced CFR correlates with myocardial hypertrophy and stress testing reveals diminished CFR even at rest, contributing to angina, dyspnea, accelerated fibrosis and increased arrhythmia risk [38, 39]. Myocardial hypertrophy and intramural arterial wall remodeling trigger ischemia, leading to the release of profibrotic cytokines such as TGF-β, which worsens endothelial dysfunction, depletes NO bioavailability [40, 41]. This forms a pathological "ischemia-hypertrophy-fibrosis" feedback loop that is notoriously difficult to reverse with conventional single-target agents Scheme 1.

HYQT, an in-house formulation patented by the Affiliated Hospital of Liaoning University of Traditional Chinese Medicine. ISO, a β-adrenergic agonist, induces HCM-like models by activating the cyclic adenosine monophosphate–protein kinase A pathway, triggering excessive myocardial contraction and remodeling that mimic human HCM pathology [42]. Continuous ISO administration induced cardiac enlargement, hypertrophic marker upregulation (ANP, BNP, NT‑proBNP), impaired systolic function, and electrophysiological abnormalities, which were reversed by HYQT in a dose-dependent manner. The high-dose group (32 g/kg) exhibited significant suppression of cardiac hypertrophy, with efficacy comparable to the positive control, nicorandil. However, beyond symptomatic effects, our study provides mechanistic insight into how HYQT modulates key pathological processes associated with this feedback loop, through coordinated regulation of cardiomyocyte remodeling and microvascular function.

Alpha-smooth muscle actin (α-SMA), a marker of myofibroblasts, drives fibroblast proliferation and type I collagen deposition in HCM, leading to myocardial stiffness, impairing diastolic function and elevating arrhythmia risk [43]. HCM progression is associated with reduced eNOS expression, increased oxidative stress (elevated MDA, decreased T-SOD, CAT, and GSH), and elevated pro-inflammatory cytokines (TNF-α, IL-1β, IL-6), promoting fibrosis [44, 45]. Microvascular dysfunction, caused by fibrotic remodeling and endothelial dysfunction, exacerbates ischemia and inflammation, contributing to perfusion heterogeneity. By therapeutically intervening in this complex microenvironment, HYQT reduced α-SMA and collagen I expression, alleviating myocardial stiffness. It inhibited iNOS, restored eNOS, and improved NO bioavailability, promoting vasodilation. HYQT also decreased MDA and enhanced antioxidant enzyme activities (T-SOD, CAT). Furthermore, HYQT enhanced myocardial perfusion, restored eNOS expression, enhanced VEGF-mediated angiogenesis, and upregulated ZO-1 to improve vascular barrier integrity. It suppressed pro-inflammatory cytokines and increased IL-10, reducing inflammation. These findings demonstrate HYQT’s holistic ability to concurrently mitigate cardiac hypertrophy, inflammation, fibrosis, oxidative stress, and microvascular dysfunction, supporting its potential as a therapeutic for HCM.

This study robustly demonstrates HYQT's potent efficacy against HCM. However, its precise mechanisms are challenging to pinpoint due to the heterogeneous composition of TCM, with multiple bioactive components targeting diverse pathways. In the present study, UHPLC–MS analysis identified 31 absorbed HYQT compounds, including flavonoids (e.g., baicalin, apigenin glucuronide), terpenoids (e.g., cryptotanshinone, tanshinone IIA), and saponins (e.g., formononetin glycoside). These multi-class compounds provide the material basis for TCM's synergistic action.

Network pharmacology revealed 150 overlapping targets between HYQT compounds and HCM-related pathways, with GO enrichment highlighting MAPK regulation and apoptosis. KEGG analysis identified key pathways such as AGE–RAGE, MAPK, and IL-17. Molecular docking confirmed that HYQT components, especially baicalin and apigenin glucuronide, interact with MAPK1/p38 MAPK, supporting MAPK inhibition. In HCM, microvascular impairment stems partly from HIF-1α dysregulation during capillary growth; restoring HIF-1α enhances capillary density and function [46–48]. Our docking results further suggested that HYQT constituents, such as cryptotanshinone and tanshinone IIA, exhibit favorable binding affinity to HIF-1α. This computational evidence supports a potential bifurcated regulatory mechanism, whereby HYQT may concurrently suppress MAPK overactivation to attenuate cardiomyocyte remodeling and enhance HIF-1α signaling to improve microvascular repair.

To validate these computational predictions, we assessed protein expression in myocardial tissue from ISO-induced HCM mice. As expected, phosphorylated MAPK1 and p38 MAPK were significantly elevated, indicating MAPK pathway activation, while HIF-1α expression was reduced, consistent with impaired microvascular function. HYQT intervention dose-dependently inhibited MAPK phosphorylation and restored HIF-1α levels.

To further examine cell-type–specific regulation, we established parallel in vitro injury models using H9C2 cardiomyocytes and HCMECs. In HCMECs, HYQT-containing serum improved migration and viability in a dose-dependent manner, suggesting a role in promoting vascular repair through VEGF-mediated angiogenesis. Concurrently, in H9C2 cells, HYQT reduced oxidative stress and inhibited MAPK phosphorylation, thereby attenuating hypertrophic responses.

To strengthen mechanistic inference beyond correlative observations, we incorporated pharmacological inhibitors for pathway perturbation. In H9C2 cardiomyocytes, co-treatment with MAPK pathway inhibitors further enhanced the anti-hypertrophic and antioxidant effects of HYQT, as evidenced by additional reductions in ANP, BNP, and intracellular ROS levels, supporting the involvement of MAPK signaling in HYQT-mediated protection. In HCMECs, pharmacological inhibition of HIF-1α using YC-1 attenuated the HYQT-induced upregulation of CD31 and VEGF and diminished improvements in cell viability and ROS-related parameters, indicating that the endothelial protective effects are at least partially dependent on HIF-1α–related signaling. Collectively, these findings provide pharmacological evidence that HYQT exerts cell-type-specific regulatory effects by modulating MAPK signaling in cardiomyocytes and HIF-1α–mediated pathways in endothelial cells.

Although MAPK and HIF-1α are well-established signaling pathways, this study highlights their multi-cellular modulation by the clinical TCM formula HYQT. This regulation is associated with improvements in both myocardial hypertrophy and coronary microvascular dysfunction in hypertrophic cardiomyopathy. The integrated modulation of these pathways across cardiomyocytes and microvascular endothelial cells remains insufficiently characterized, and our findings provide a cell-type–resolved perspective on this process. Collectively, these results support a multi-pathway, multi-target regulatory framework, reflecting the complex and system-level characteristics of TCM.

This study has several limitations. Systemic omics analyses such as transcriptomic sequencing (RNA-seq) were not performed, and thus we were unable to globally screen all potential regulatory pathways associated with the observed phenotypic improvements at the transcriptome level. In future work, we will further integrate RNA-seq–based omics analysis with phenotype-correlation strategies to systematically and unbiasedly characterize the core regulatory networks underlying phenotypic improvement, providing more comprehensive omic evidence for mechanistic interpretation.

## Conclusion

This study provides mechanistic insight into the therapeutic effects of HYQT in hypertrophic cardiomyopathy. UHPLC-MS identified 31 active components, supporting its pharmacological basis. HYQT modulates two parallel pathways: it inhibits MAPK phosphorylation to attenuate myocardial hypertrophy and fibrosis, while enhancing HIF-1α activity to promote angiogenesis. These effects were supported by network pharmacology, molecular docking, and Western blot analysis. In vivo, HYQT improved cardiac function, attenuated fibrosis, and restored oxidative balance. In vitro, HYQT-containing serum promoted endothelial migration, reduced ROS levels, and enhanced cell viability. High-dose HYQT did not show signs of hepatotoxicity or nephrotoxicity. Importantly, our findings highlight a coordinated, cell-type–specific regulatory effect, involving both cardiomyocytes and microvascular endothelial cells. Collectively, these multi-target effects are associated with mitigation of the complex pathophysiology of HCM, suggesting that HYQT may serve as a promising therapeutic strategy.

## Supplementary Information

Supplementary Material 1.

Supplementary Material 2.

## Data Availability

The core data of this work are provided in the manuscript. Further information will be provided upon reasonable request. No datasets were generated or analysed during the current study.

## Abbreviations

HCM: Hypertrophic cardiomyopathy
CMD: Coronary microvascular dysfunction
HYQT: Huayu Qutan Formula
ISO: Isoproterenol
HCMECs: Human cardiac microvascular endothelial cells
ROS: Reactive oxygen species
MAPK: Mitogen-activated protein kinase
HIF-1α: Hypoxia-inducible factor 1-alpha
VEGF: Vascular endothelial growth factor
ANP: Atrial natriuretic peptide
BNP: B-type natriuretic peptide
NT-proBNP: N-terminal pro-B-type natriuretic peptide
MDA: Malondialdehyde
TNF-α: Tumor necrosis factor-alpha
IL-6: Interleukin-6
ERK: Extracellular signal-regulated kinase
JNK: C-Jun N-terminal kinase
T-SOD: Total superoxide dismutase
CAT: Catalase
NO: Nitric oxide
T-NOS: Total nitric oxide synthase
iNOS: Inducible Nitric Oxide Synthase
GSSG: Oxidized Glutathione
T-GSH: Total Glutathione
IL-1β: Interleukin-1beta
IL-10: Interleukin-10
H&E: Hematoxylin and Eosin
α-SMA: α-Smooth muscle actin
ZO-1: Zonula occludens-1
CCK-8: Cell Counting Kit-8
LV: Left ventricular
IVSd/IVSs: Interventricular septal thickness (diastolic/systolic)
LVPWd/LVPWs: Left ventricular posterior wall thickness (diastolic/systolic)
LVMass: Left ventricular mass
LVIDd/LVIDs: Left ventricular internal diameter at end-diastole/end-systole
EF: Ejection fraction
FS: Fractional shortening
HW/BW: Heart weight to body weight ratio
ECG: Electrocardiogram
ALT: Alanine aminotransferase
AST: Aspartate aminotransferase
ALP: Alkaline phosphatase
ALB: Albumin
UREA: Blood urea nitrogen
CREA: Creatinine
TGF-β: Transforming growth factor-β
