# Supplementary figures and images for: Huayu-qutan formula ameliorates hypertrophic cardiomyopathy by regulating MAPK and HIF‑1α-signaling pathways
Source: Chin Med. 2026 May 29;21:154. doi: 10.1186/s13020-026-01400-5 (PMC13220444; doi:10.1186/s13020-026-01400-5)

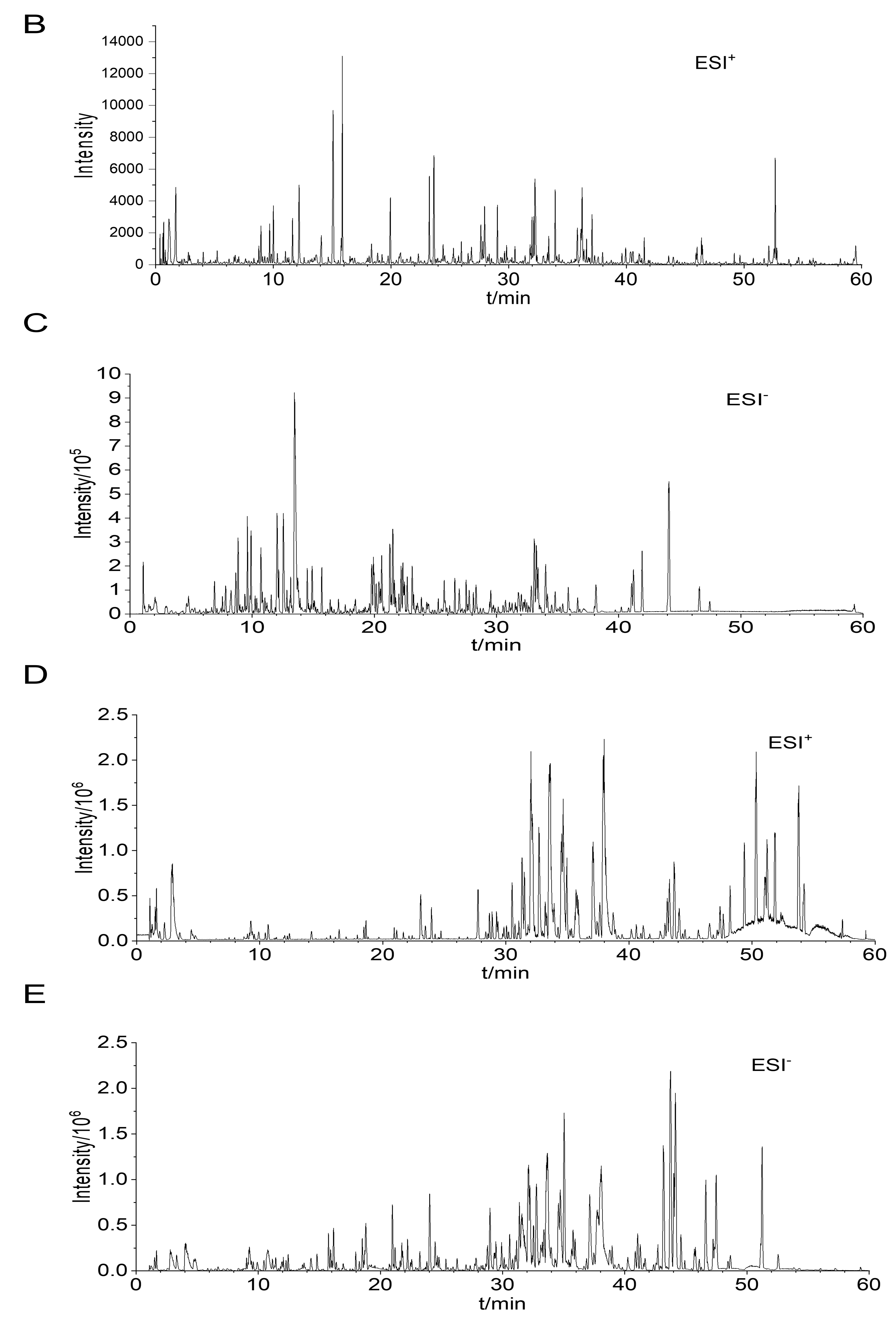


Fig.6


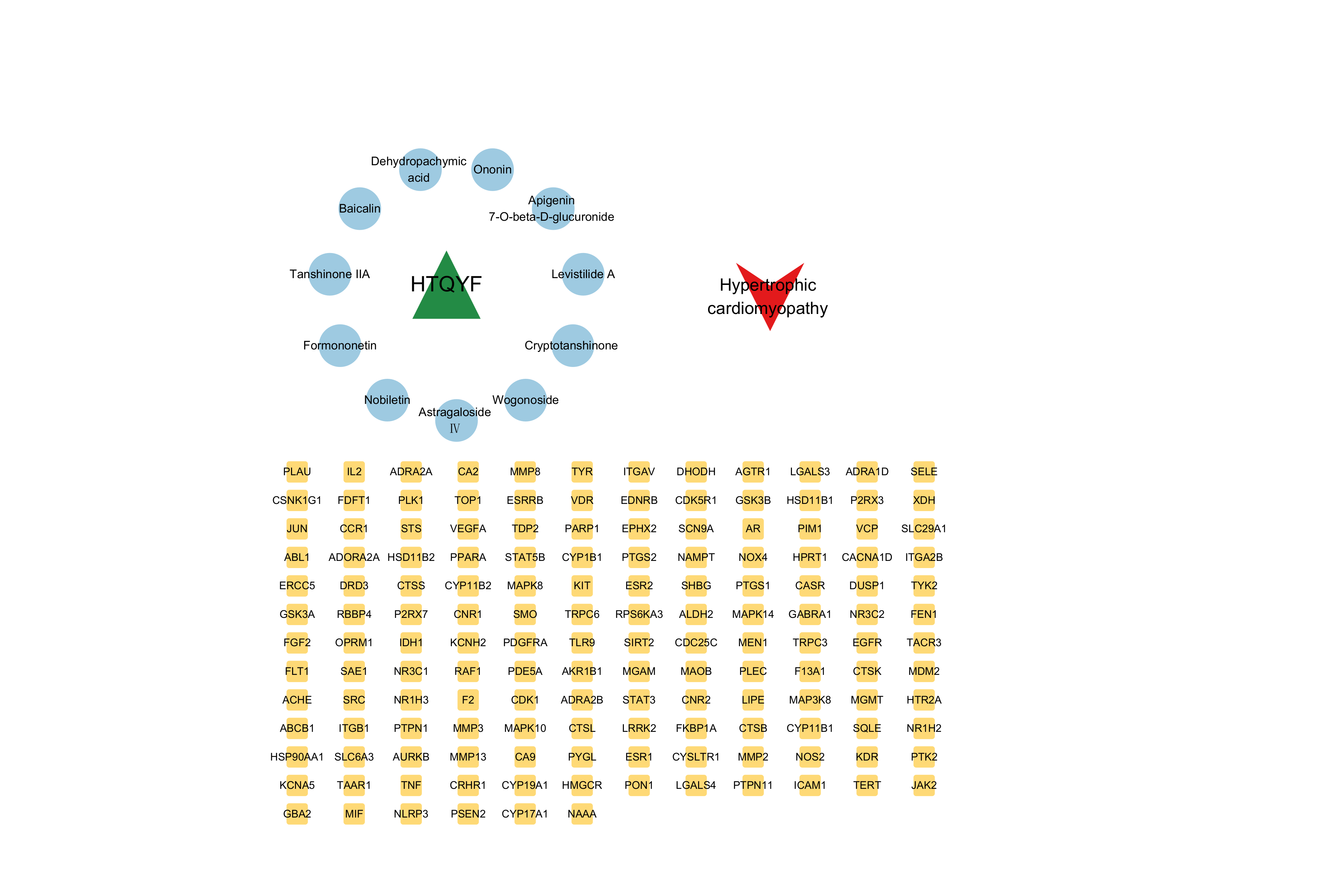


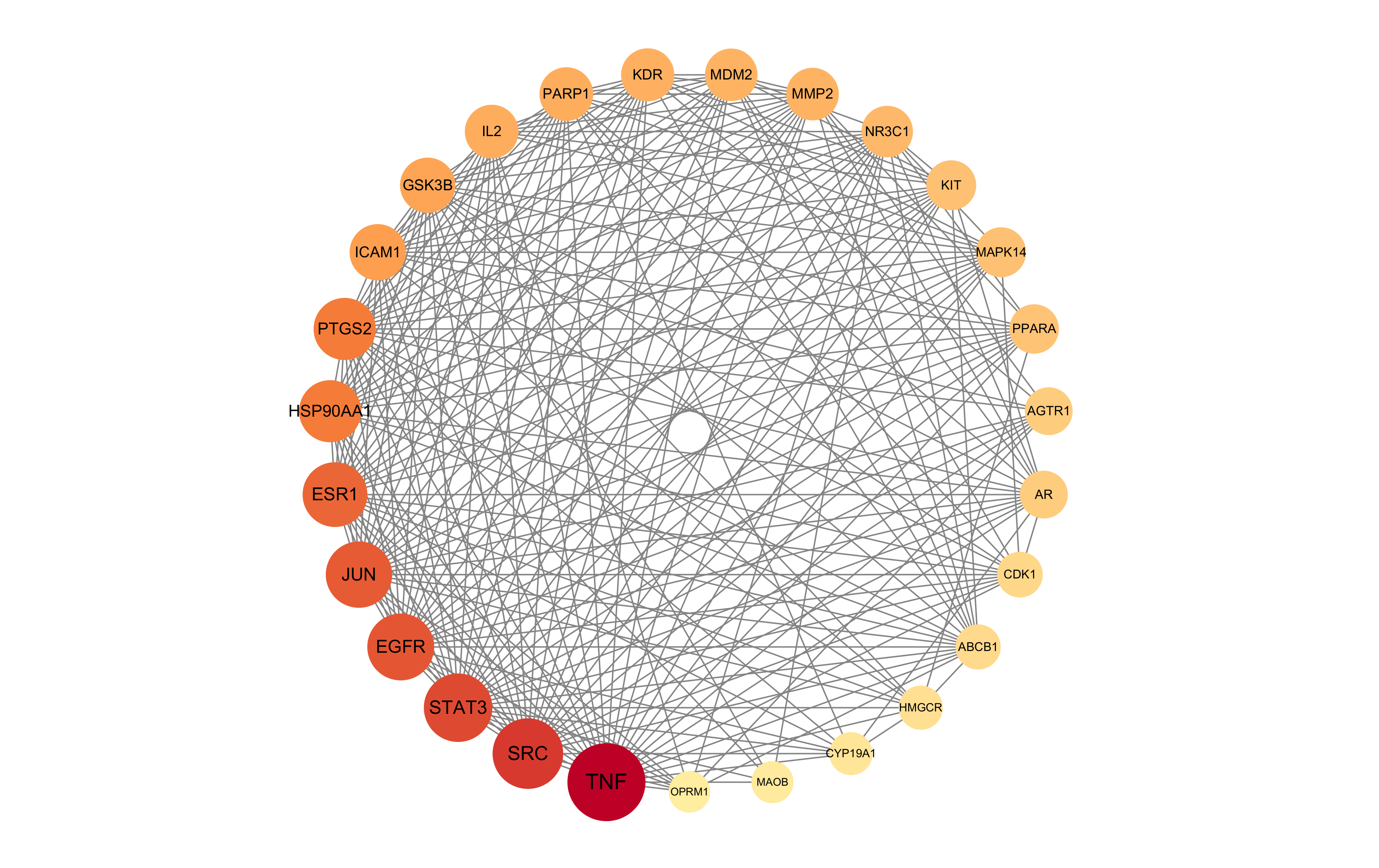



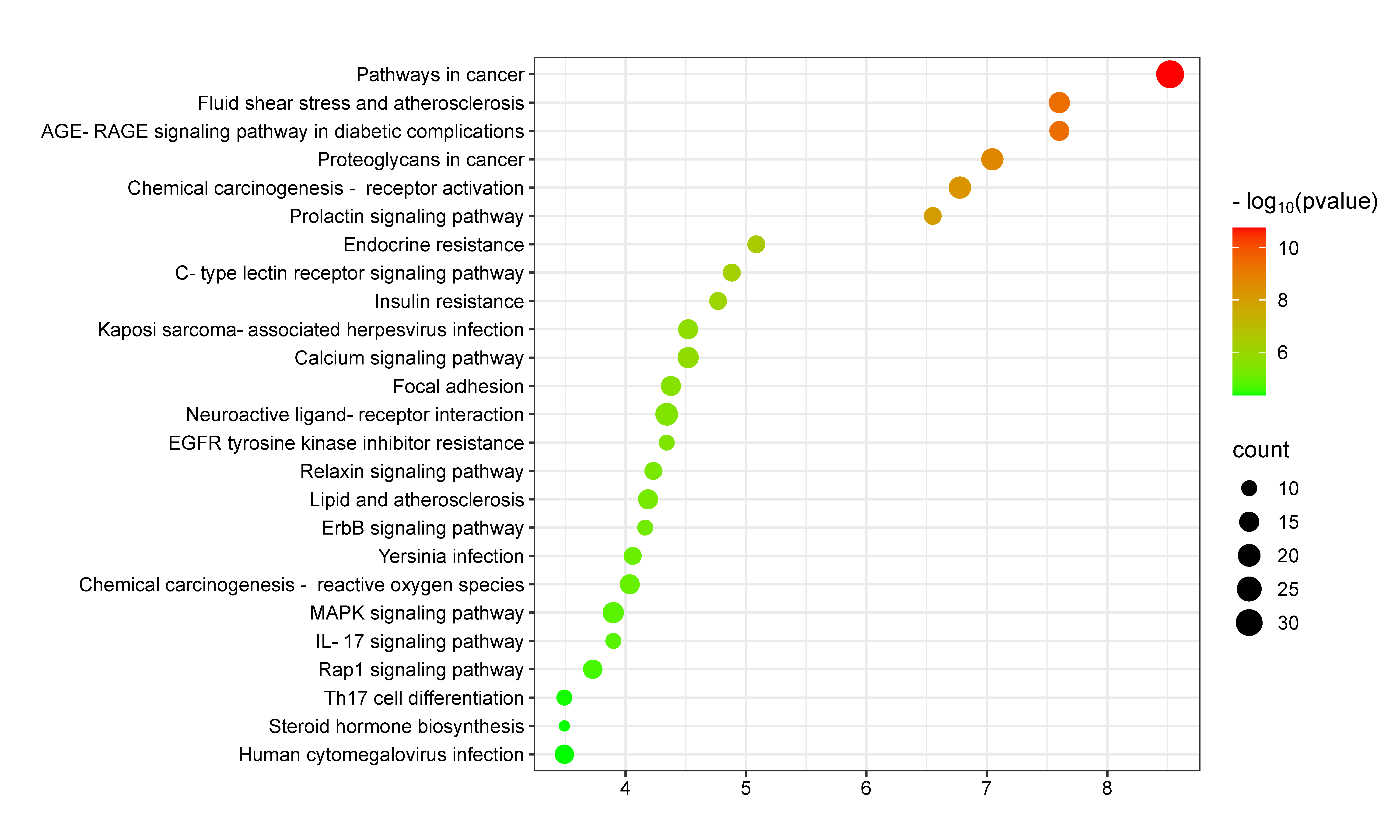


Fig.6

Supplement: Supplementary file 2 — Supplementary Material 2. [file 13020_2026_1400_MOESM2_ESM.doc]
